# Proteomic analysis of dorsal root ganglia in a mouse model of paclitaxel-induced neuropathic pain

**DOI:** 10.1371/journal.pone.0306498

**Published:** 2024-09-27

**Authors:** Rania Hanna, Alexandru Graur, Patricia Sinclair, Bryan D. Mckiver, Paula D. Bos, M. Imad Damaj, Nadine Kabbani

**Affiliations:** 1 Interdisciplinary Program in Neuroscience, George Mason University, Fairfax, VA, United States of America; 2 School of Systems Biology, George Mason University, Fairfax, VA, United States of America; 3 Department of Pharmacology & Toxicology, Virginia Commonwealth University, Richmond, VA, United States of America; 4 Department of Pathology, Massey Comprehensive Cancer Center, Virginia Commonwealth University School of Medicine, Richmond, VA, United States of America; ShanghaiTech University, CHINA

## Abstract

Paclitaxel is a chemotherapy drug widely used for the treatment of various cancers based on its ability to potently stabilize cellular microtubules and block division in cancer cells. Paclitaxel-based treatment, however, accumulates in peripheral system sensory neurons and leads to a high incidence rate (over 50%) of chemotherapy induced peripheral neuropathy in patients. Using an established preclinical model of paclitaxel-induced peripheral neuropathy (PIPN), we examined proteomic changes in dorsal root ganglia (DRG) of adult male mice that were treated with paclitaxel (8 mg/kg, at 4 injections every other day) relative to vehicle-treated mice. High throughput proteomics based on liquid chromatography electrospray ionization mass spectrometry identified 165 significantly altered proteins in lumbar DRG. Gene ontology enrichment and bioinformatic analysis revealed an effect of paclitaxel on pathways for mitochondrial regulation, axonal function, and inflammatory purinergic signaling as well as microtubule activity. These findings provide insight into molecular mechanisms that can contribute to PIPN in patients.

## Introduction

Paclitaxel is a taxane class compound used as a cytotoxic chemotherapy agent in the treatment of various solid tumors including prostate, ovarian, breast cancer, bladder, esophageal, small and non-small-cell lung, pancreas, and melanoma [1, 2]. Chemotherapy, with compounds such as paclitaxel, may interfere with a patient’s course of treatment due to the emergence of pain symptoms in over 50% of cases [3]. There is no effective mechanism for the prevention of chemotherapy associated neuropathy and conventional analgesics and other pain medications are generally ineffective in negative symptom management [4].

Paclitaxel acts by disrupting proliferation of cancer cells through binding with β-tubulin [5] and suppressing its depolymerization. However, paclitaxel can also target non-cancer cells leading to a number of side effects including skin toxicity like rash and pruritus [6], and neuronal toxicity associated with peripheral neuropathy [7]. Studies show that paclitaxel treatment causes changes in mitochondrial function, increased inflammation within dorsal root ganglia (DRG) and an increase in proapoptotic factors [7, 8].

The DRG is a primary sensory module consisting of sensory neurons, satellite glia, and immune cells such as macrophages and T cells [9]. Large DRG gives rise to myelinated axons that are involved in mechanoreception, whereas smaller DRG give rise to unmyelinated axons, which are involved in mechanoreception, thermoreception, and nociception [10]. Paclitaxel has been shown to traverse the blood-nerve barrier surrounding the DRG and bind microtubules within neurons, causing damage to axons and nerve fibers [11]. Microtubule stabilization underlies axonal transport loss, which promotes axonal degeneration, leading to peripheral neuropathy [12]. Paclitaxel induced peripheral neuropathy (PIPN) is associated with damage to sensory axons and nerve fibers in humans and animal models [13, 14].

Animal models exist to study molecular changes within sensory systems responding to paclitaxel exposure. Transcriptomic analysis of the DRG suggests an important role for paclitaxel in the activation of neuroinflammatory signaling within mice [14–16] however less is known about proteomic changes. In this study, we examined proteomic changes that may contribute to PIPN in a pre-clinical mouse model with heightened pain-like behaviors [17]. Specifically, the current proteomic analysis is conducted in mice that exhibit paclitaxel-induced mechanical hypersensitivity and cold hypersensitivity, as well as a decreased sensory nerve compound action potential (SNCAP) [17]. Our proteomic analysis aims to provide knowledge on mechanistic drivers of neuropathy during paclitaxel treatment.

## Methods

### Animals

The experiments were performed on 12-week-old male C57BL/6J mice (Strain #000664, The Jackson Laboratory, Bar Harbor, ME). Animals were housed in groups of 4 per cage with enriched environment and maintained on a 12 hour light/dark cycle, at a 22°C room temperature with ad libitum access to food (global 18% protein chow diet; Envigo Teklad, Indianapolis, IN, USA) and water. DRG from 8 animals for both the experimental and control groups were used, with 3 mass spectrometry technical replicates completed.

### Ethical statement

All animal experiments were performed under an approved IACUC protocol in the Division of Animal Research of Virginia Commonwealth University (Richmond, VA), accredited by the Association for Assessment and Accreditation of Laboratory Animal Care (AALAC). All efforts were made to reduce the number of animals used in this study and to ensure optimal conditions of well-being before, during and after each experiment. Mice were observed daily for general well-being and their weight was measured weekly. All behavioral experiments were performed during the light cycle and with the observer unaware of the experimental treatment of the animals.

### Drug treatment

Paclitaxel was purchased from VCU Health Pharmacy (Athenex, NDC 70860-200-50, Richmond, VA, USA) and dissolved in a 1:1:18 mixture of 200 proof ethanol, kolliphor, and distilled water (Sigma-Aldrich). Paclitaxel was administered at a dose of 8 mg/kg intraperitoneally (i.p.) every other day; 4 administrations completed one regimen. Control mice received a10 ml/kg i.p. injection of the diluent, with the same injection regimen. Seven days after drug treatment, mice were euthanized by decapitation. Lumbar (L4-L6) DRG tissue was collected, immediately frozen in liquid nitrogen, and stored at -80°C until analysis.

### Protein isolation

DRG cytoplasmic and membrane enriched protein fractions were obtained as described in [18]. Briefly, DRG specimens were combined with 500 μL of lysis buffer A (NaCl, HEPES, digitonin, hexylene glycol, protease, and phosphatase inhibitor cocktail) and disrupted for 5 seconds using a dounce homogenizer. The resulting tissue suspension was processed with a QIAshredder homogenizer (Qiagen, 79656) and centrifuged at 500g for 10 minutes to filter the homogenate. The pellet was resuspended in 500 μL of lysis buffer A, incubated on a nutator for 10 minutes and centrifuged at 4000g for 10 minutes to isolate the cytosolic proteins. The pellet was resuspended in 1 mL of lysis buffer B (NaCl, HEPES, Igepal, hexylene glycol, protease, and phosphatase inhibitor cocktail), incubated for 30-minutes on a nutator, then centrifuged at 6000g for 10 minutes to collect membrane bound proteins. All centrifugation and incubation steps were performed at 4°C.

### Liquid-chromatography electrospray ionization mass spectrometry

Liquid-chromatography electrospray ionization mass spectrometry (LC-ESI MS/MS) was conducted in data-dependent acquisition (DDA) mode similar to previous studies [19, 20]. Briefly, proteins were precipitated by incubating for 5-minutes in acetone on ice followed by centrifugation. Proteins were denatured, reduced, and alkylated in 8 M urea, 1 M dithiothreitol, and 0.5 M iodoacetamide. Proteins were digested in trypsin (0.5 μg/μl) in 500 mM ammonium bicarbonate at 37°C for 5 hours and the fragments were desalted with C-18 ZipTips (Millipore), dehydrated in a SpeedVac for 18 mins, and reconstituted in 0.1% formic acid.

An Exploris Orbitrap 480 equipped with an EASY-nLC 1200 HPLC system (Thermo Fisher Scientific, Waltham, MA, USA) was used to conduct LC-ESI MS/MS analysis. Peptide separation was accomplished using a reverse-phase PepMap RSLC 75 μm i.d by 15 cm long with a 2 μm particle size C18 LC column (Thermo Fisher Scientific, Waltham, MA, USA). A solution of 80% acetonitrile and 0.1% formic acid was used for the peptide elution step at a flow rate of 300 nl/min. A full scan at 60,000 resolving power from 300 m/z to 1200 m/z was followed by peptide fragmentation with high-energy collision dissociation (HCD) at a normalized collision energy of 28%. EASY-IC filters were enabled for internal mass calibration, monoisotopic precursor selection, and dynamic exclusions (20 s). Data were recorded for peptide precursor ions with charge states ranging from +2 to +4. All samples were run in 3 technical replicates.

### Proteomic and statistical analysis

Proteins were identified using the SEQUEST HT search engine within Proteome Discoverer v2.4 (Thermo Fisher Scientific, Waltham, MA, USA). Raw MS peptide spectra were compared to the NCBI mouse protein database using specific search engine parameters: mass tolerance for precursor ions of 2 ppm; mass tolerance for fragment ions of 0.05 Da; and a false discovery rate (FDR) cut-off value of 1% for reporting peptide spectrum matches (PSM) to the database. Peptide abundance ratios were calculated by precursor ion quantification in Proteome Discoverer v2.4, using the vehicle control group as the denominator. Statistically significant abundance ratios with adjusted p-values < 0.05 were determined using a Student’s t-test. Analysis was performed on proteins with a quantifiable spectra signal profile observed in at least 2 of the 3 technical replicates.

### Bioinformatics

Gene ontology (GO) analysis was conducted in the Database for Annotation, Visualization, and Integrated Discovery (DAVID) [21, 22]. The clustering of protein data was conducted using the Search Tool for the Retrieval of Interacting Genes/Proteins (STRING, v11.5) database using a Markov Cluster Algorithm (MCL) with an inflation parameter of 2 [23]. Data was processed, analyzed, and presented using Excel and the following tools: the R statistical software [24] and packages: ggplot2 [25], tidyverse [26].

## Results

### DRG proteomics characterization in a mouse model of paclitaxel-induced neuropathy

**Fig 1:**
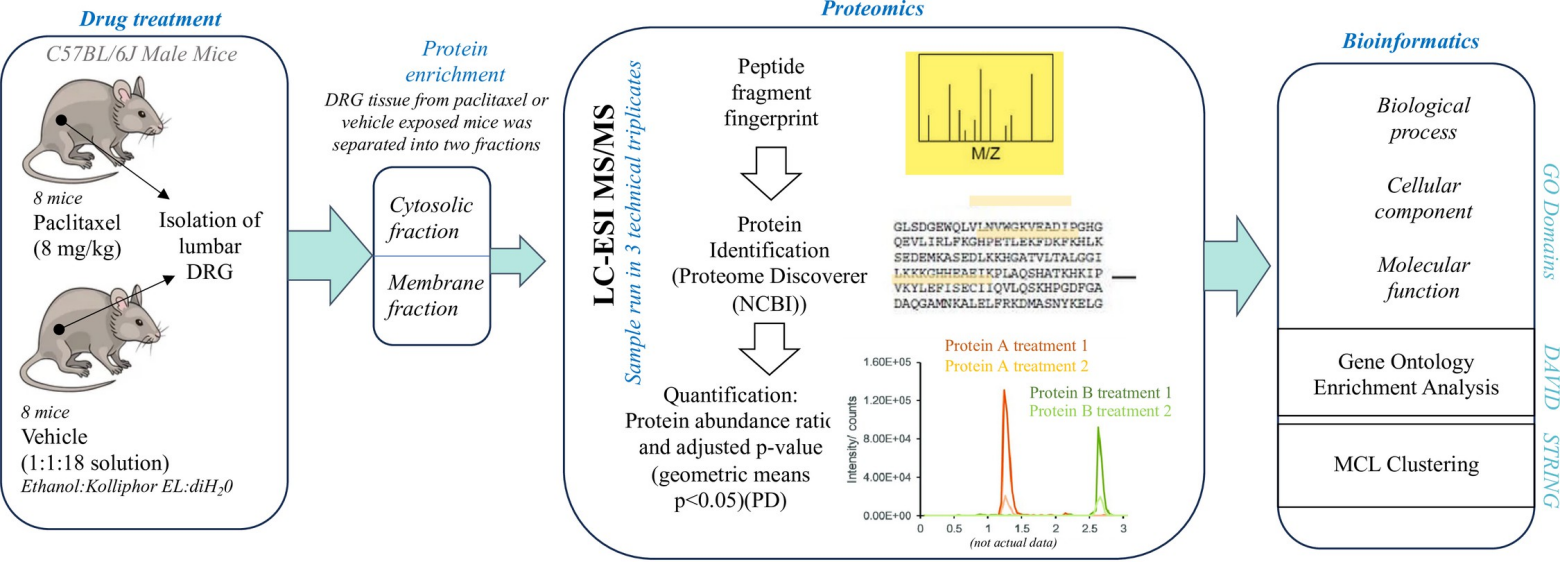
A workflow schematic showing the study design.

Paclitaxel and other chemotherapy drugs elicit peripheral nerve fiber dysfunction and neurodegeneration that drives PIPN [7]. A few rodent models exist for the study of PIPN including ours [27–29]. In recent studies, we have shown that paclitaxel administration drives altered behaviors including affective states and nociceptive responses such as mechanical allodynia and thermal hyperalgesia within mice [17, 30–34]. To understand the mechanism that may underlie these sensory responses, we assessed proteomic changes from the DRG in response to paclitaxel treatment. Eight adult male mice were treated with either 8 mg/kg body weight paclitaxel every other day for 4 days or 10 ml/kg of the vehicle diluent at the same injection regimen. Whole DRG were obtained from lumbar region L4-L6, an important site for sensory and pain processing [17]. Protein enrichment for cytosolic and membrane proteins was conducted prior to MS analysis. The overall workflow detailing the study is illustrated in **Fig 1**.

**Fig 2:**
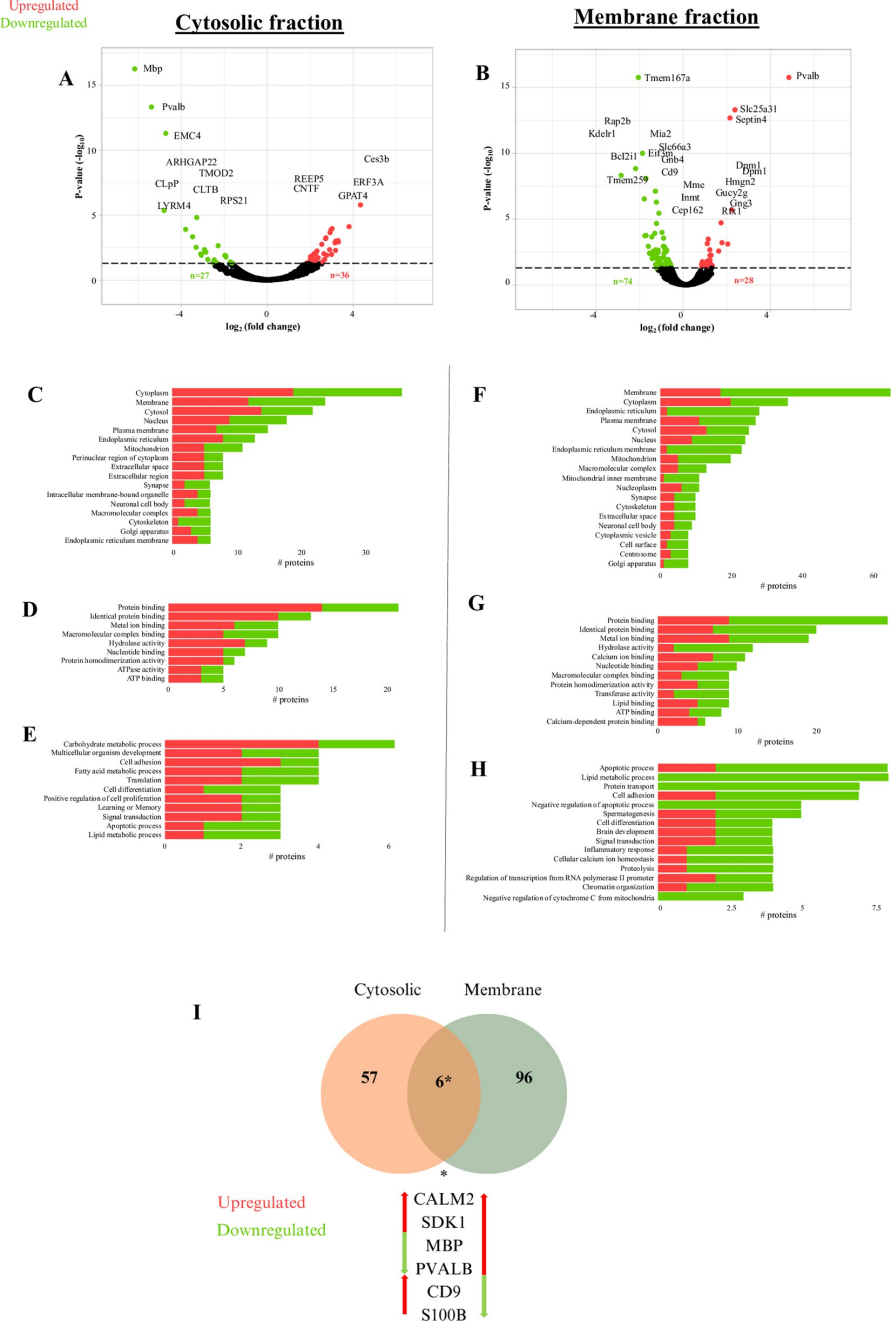
Effect of paclitaxel on the DRG proteome. Volcano plot distribution of significantly altered proteins within the DRG. A) Cytosolic fraction. B) Membrane fraction. GO terms associated with significantly altered proteins in paclitaxel treated mice relative to controls. Cytosolic protein fraction cellular component (C), molecular function (D), biological process. (E). Membrane protein fraction cellular component (F), molecular function (G), biological process (H).

Proteomic analysis of DRG was conducted in order to compare changes in protein expression using label free peptide abundance analysis, as previously shown [19, 20]. In these experiments fractions enriched for cytosolic and membrane proteins obtained from the DRG of vehicle treated mice were used as the control. Using LC-ESI MS/MS, we identified a total of 2055 proteins within the cytosolic fraction, and 2676 within the membrane fraction. These proteins are listed in **S1 File**. Statistical analysis of the abundance ratios indicates an effect of paclitaxel on 102 proteins within the membrane fraction and 63 proteins within the cytosolic fraction. The list of significantly altered proteins from each cytoplasmic and membrane fraction is presented in **S1 File**. Of the significantly altered proteins, 36 proteins are upregulated while 27 proteins are downregulated in the cytosolic fraction (**Fig 2A**). In addition, 28 proteins were upregulated while 74 are downregulated in the membrane fraction (**Fig 2B**)

We compared our proteomic dataset to published proteomic studies from mouse DRG [35–37]. 88% of proteins identified within our DRG analysis also appear in the DRG proteome of adult mice within pain related studies [37]. Overlapping DRG proteins are shown in **S1 File** [38]. A GO analysis of the significantly altered proteins indicates enrichment of cellular components, molecular functions and biological processes across cytosolic and membrane fraction proteins in the DRG of paclitaxel-treated mice (**Fig 2**). Specifically, cytosolic fraction results indicate an effect of paclitaxel on carbohydrate metabolic processes, lipid metabolism, ATPase function, cytoskeletal intermediate filament and cell adhesion, and cell proliferation as well as apoptosis (**Fig 2C–2E**). Membrane fraction results show an effect of paclitaxel on ATP activity, cell adhesion, cytoskeletal organization, protein transport, and calcium and lipid signaling (**Fig 2F–2H**). GO results appeared to slightly differ however between the two-protein fraction results with cytosolic proteomes highlighting an increase in carbohydrate metabolic processes, while the membrane fraction underscores an effect of paclitaxel on lipid metabolism. GO informatic analysis of the DRG proteome shows an effect of paclitaxel on several important metabolic pathways as well as signaling and structural functions within the DRG.

### An effect of paclitaxel on DRG energy, cytoskeletal regulation, and sensory axon signaling

We used DAVID [22, 39] enrichment analysis of GO terms that are associated with significantly altered proteins in both cytosolic and membrane fractions to compare the effect of paclitaxel on DRG proteome adaptation. As shown in **Tables 1 and 2**, enrichment analysis confirms representation of membrane and cytosolic terms within the membrane and cytosolic fractions, respectively. Additionally, enrichment analysis of the membrane fraction indicates a high representation of membrane proteins from various intracellular organelles including the mitochondria as well as the endoplasmic reticulum (ER). Membrane associated proteins are also involved in lipid, metabolic and calcium cell signaling (**Table 2**). We assessed for overlap in significantly altered proteins across the membrane and cytosolic fractions. A subset of significantly altered proteins appeared in both membrane and cytosolic fractions results (**Fig 2I**). These proteins include cluster of differentiation 9 (CD9), myelin basic protein (MBP), calcium binding proteins S100B, parvalbumin (Pvalb),calmodulin, and the cell adhesion molecule SDK1. An analysis of the effect of paclitaxel reveals that some of these overlapping proteins are differentially regulated by the drug treatment condition (**Fig 2I**). These findings suggest an effect of paclitaxel on specific functional proteins within the DRG.

**Table 1:** DAVID enrichment terms and their associated proteins within the cytosolic fraction.

GO category	Term	Count	Proteins	Adjusted p-value
Cellular component	Cytoplasm	36	YWHAE, MTPN, CNTF, TAGLN, GSPT1, CST3, ARHG AP22, HEBP2, MBP, PTGDS, GET3, EIF4E, PHM1, APC2, TMP3, TMOD2, CDC42BPB, TBCA, S100B, TSN, KIF27, PPP1CA, CARHSP1, RPS28, PSMA4, STK24, NACA, SLMAP, GPD1, G6PDX, GNB3, NAA15, GPD1L, CALM2, RPS21, PVALB	0.009819583
Molecular function	Macromolecular complex binding	10	YWHAE, CNTF, TTR, VAPA, CDC42BPB, ATP5F1D, TSN, ATP5F1E, PVALB, PPP1CA	0.027691179

**Table 2:** DAVID enrichment terms and their associated proteins within the membrane fraction.

**GO category**	**Term**	**Count**	**Proteins**	**Adjusted p-value**
Cellular component	Membrane	64	LGALS3BP, CLIC4, GOLT1B, KDELR1, ICAM2, MIA2, ATP2A1, FNBP1L, COX7C, TMEM186, CLGN, NDST3, ANXA6, SLC36A1, ENTPD1, MME, SIGMAR1, TAP1, DYNLL1, TLCD3B, MFSD10, DPM1, TMEM256, RAP2B, STIM1, DAD1, GORASP2, TMEM259, NDUFS4, S100A6, CD47, S100A9, CERS2, CDS1, RTN3, STOML2, SLC66A3, MGST3, PON2, DERL1, SLC1A4, ASAP2, HIGD1A, GUCY2G, ZMPSTE24, MTDH, PTDSS2, GNG3, MBP, CD99, MPDUL1, S100A10, NDUFA3, SURF4, ERLINI, SDK1, TSPAN18, CD9, MAN2B1, ESYT2, SLC25A31, CALM2, TOMM5, BCL2L1, CDS2	0.00004
Cellular component	Endoplasmic reticulum	28	CDS1, RTN3, GOLT1B, KDELR1, MGST3, DERL1, MIA2, ATPSA1, CLGN, MTDH, ZMPSTE24, PTDSS2, SLC36A1, RPS7, SURF4, SIGMAR1, ERLIN1, TAP1, TLCD3B, DPM1, STIM1, DAD1, GORASP2, TMEM259, ESYT2, BCL2L1, CERS2, CDS2	0.00000035
Cellular component	Endoplasmic reticulum membrane	23	CDS1, RTN3, KDELR1, SURF4, SIGMAR1, ERLIN1, DERL1, TAP1, MIA2, ATP2A1, TLCD3B, CLGN, MTDH, ZMPSTE24, DPM1, PTDSS2, STIM1, DAD1, GORASP2, TMEM259, ESYT2, CERS2, CDS2	.000000294
Cellular component	Mitochondrion	20	STOML2, CLIC4, NDUFA3, MGST3, TAP1, ATP2A1, SEPTIN4, DYNLL1, HIGD1A, COX7C, TMEM186, TXN2, NDUFS4, ANXA6, ACOT2, PPIF, PMPCB, SLC25A31, TOMM5, BCL2L1	0.030463258
Cellular component	Macromolecular complex	13	PRPS1, RPS7, GOLT1B, ERLINI, HIGD1A, ZMPSTE24, STIM1, ANXA6, GNB4, CD9, MBP, CALM2, PVALB	0.045210595
Cellular component	Mitochondrial inner membrane	11	STOML2, TMEM256, NDUFA3, NDUFS4, PPIF, PMPCB, SLC25A31, HIGD1A, COX7C, TMEM186, BCL2L1	0.0093664559
Molecular function	Lipid binding	9	STOML2, FABP4, APOH, ERLINI, ANXA6, PMP2, ESYT2, MBP, FNBP1L	0.031904593
Molecular function	Calcium-dependent protein binding	6	ANXA6, S100A6, S100B, CALM2, S100A9, S100A10	0.016979225

**Fig 3:**
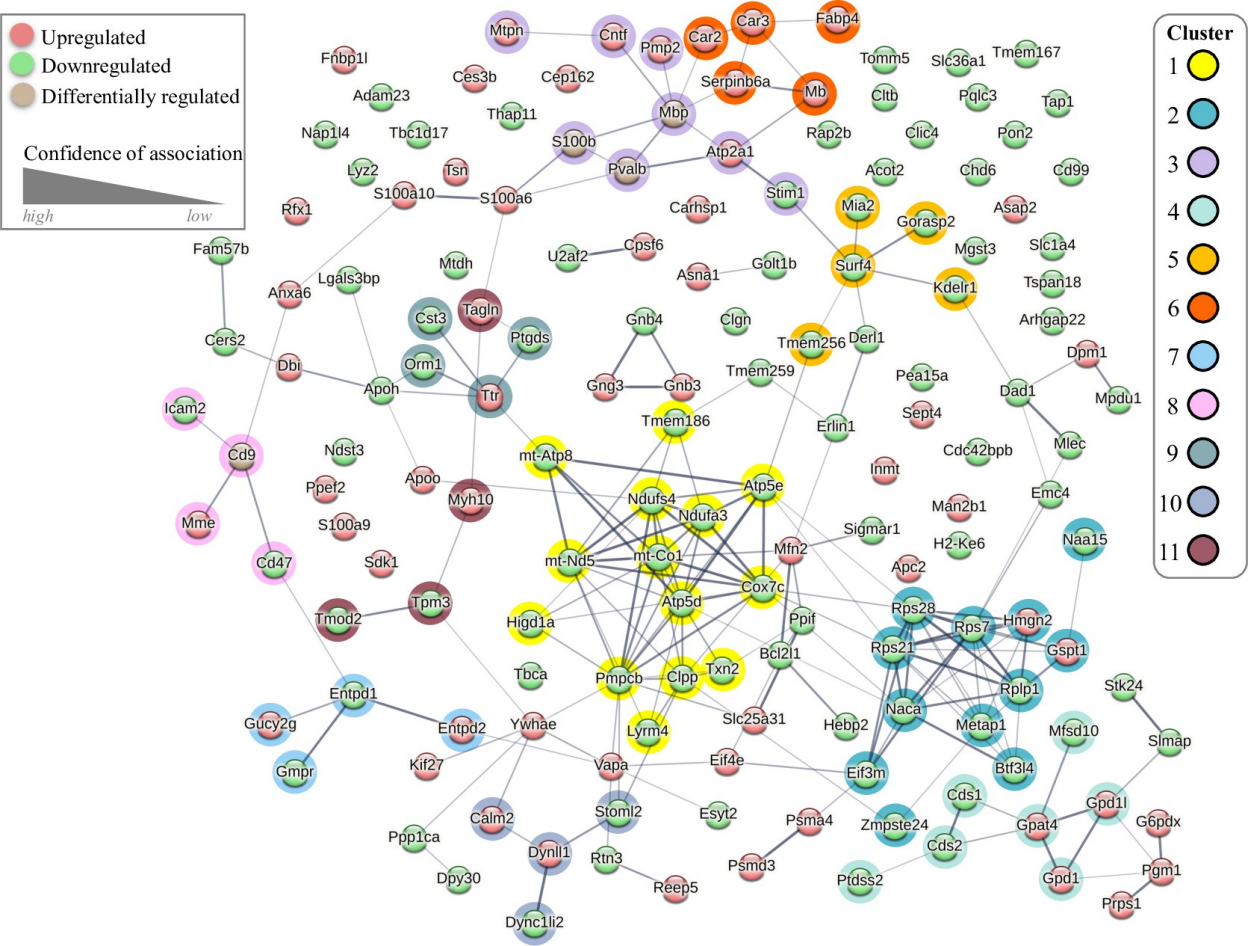
A protein-protein interaction (PPI) map of the paclitaxel associated DRG proteome. STRING network showing PPI amongst all significantly altered proteins. Protein clusters within the network are highlighted by color. The thickness of the connection indicates the degree of confidence between node associations while color indicates whether the protein is increased (red), decreased (green) or differentially altered (light brown) by paclitaxel. The STRING network is based on an MCL algorithm with an inflation parameter of 2 used to identify 11 cluster groups.

**Fig 4:**
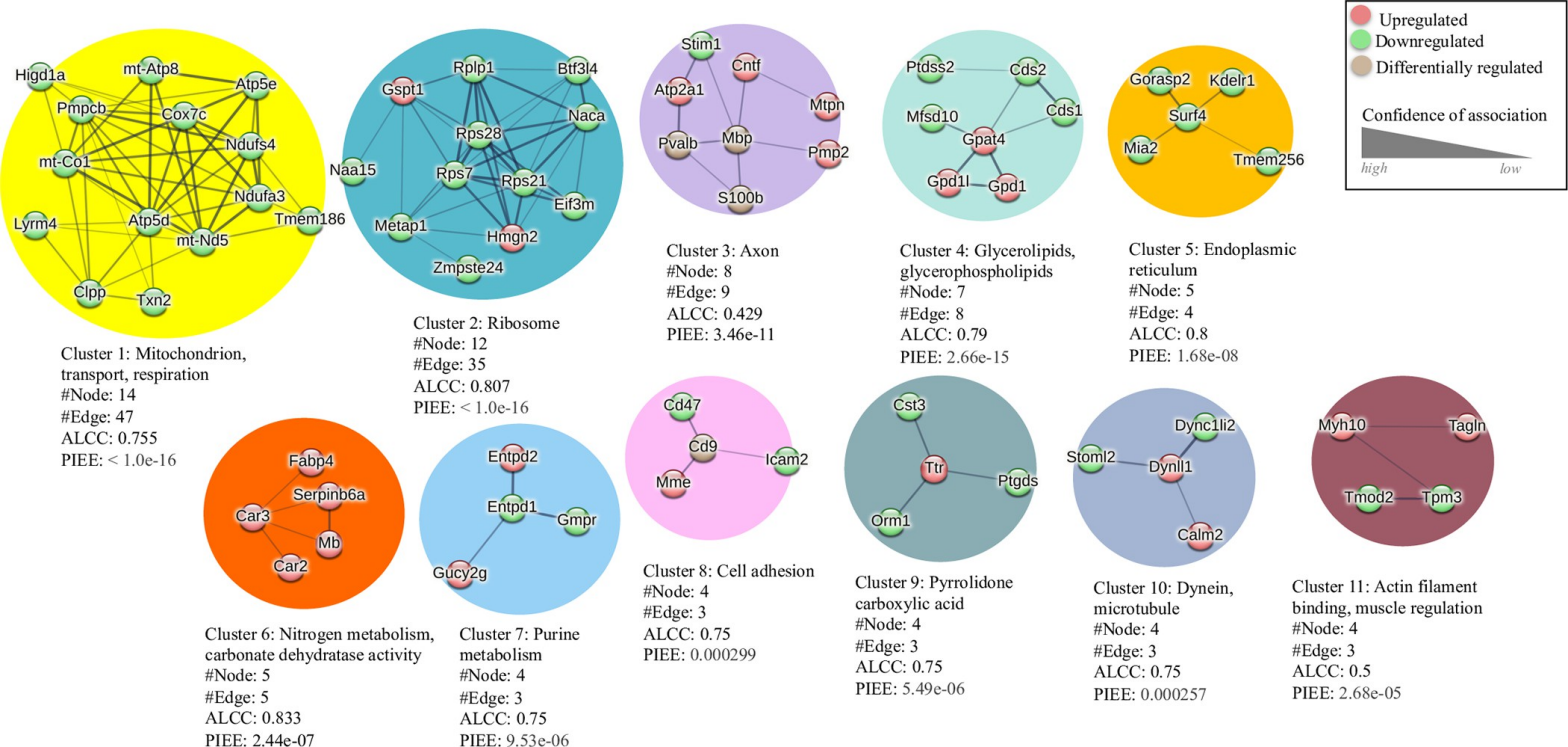
Primary cluster groups within the PPI network. An enrichment tag analysis showing the 11 cluster groups within the STRING analysis of the paclitaxel associated DRG proteome.

Proteomic analysis can also offer insight into drug-treatment associated adaptations within protein-protein interaction (PPI) networks within target tissue and cell types [40].We used a Markov cluster (MCL) analysis to define PPI networks within the DRG proteome consisting of all significantly upregulated and downregulated proteins from membrane and cytosolic fractions. MCL analysis is represented by an integrated PPI network based on the identity of the significantly altered proteins in response to paclitaxel treatment (**Fig 3**). Within the PPI network we identified 11 high confidence protein clusters. The largest PPI cluster (Cluster 1) was found to contain 14 proteins with 47 connections yielding a significant PPI connection (p < 1.0 × 10^−16^). It consists of mitochondrial proteins and is positioned centrally within the integrated PPI map (**Fig 3)**. Cluster 1 had an average local clustering coefficient (ALCC) of 0.755 with analysis revealing downregulation in this mitochondrial protein network (**Fig 4**). The second cluster (Cluster 2) consists of 12 ribosomal proteins that also appear predominantly downregulated within the DRG in response to paclitaxel (**Fig 4**). Additional clusters identified within PPI analysis include cell structural and motility regulators that include cytoskeletal elements (e.g., Clusters 8, 10, 11) and protein clusters involved in biochemical pathways including nitrogen and pyridine metabolism (Clusters 6 and 7).

## Discussion

We used a proteomic research strategy to examine molecular changes within the DRG in an established mouse model of PIPN[17, 41]. In recent studies, OMIC methods such as metabolomics, proteomics, and phospho-proteomic analyses were used to identify molecular changes in the DRG during diabetic neuropathy in humans [42]. Our study expands this methodology and assesses the specific impact of paclitaxel on the DRG proteome within a PIPN mouse model. Overall, our proteomic results highlight putative disruption to mitochondrial components and related metabolic processes within the DRG during paclitaxel administration, similar to studies that indicate that paclitaxel causes mitochondrial damage in during axonal damage [43–45]. In addition, our findings support the role of paclitaxel in the remodeling of intracellular compartments such as the ER, Golgi, and the cytoskeleton through changes in actin filament and dynein microtubule proteins [44–46]. These results provide evidence on the role of paclitaxel in nervous tissue, and shed light on the potential impact of chemotherapy on DRG associated sensory and neuroimmune activity [47, 48].

**Fig 5:**
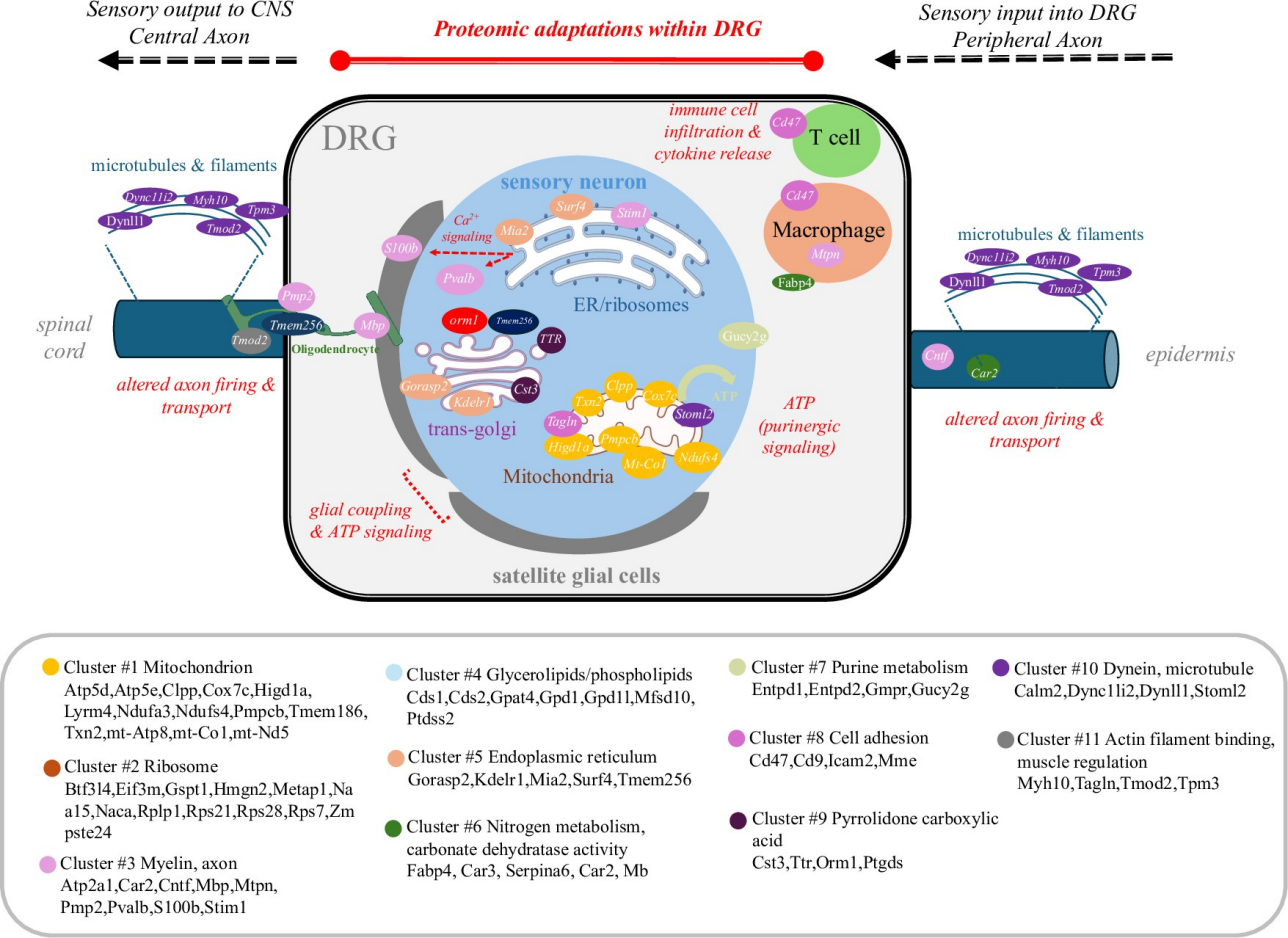
A signaling pathway hypothesis model showing the effect of paclitaxel on proteins within the DRG.

Paclitaxel binds to microtubules in both cancer and non-cancer cells impairing cell division as well as other functions. Studies in rodent models show that paclitaxel treatment can also lead to the demyelination and degeneration of axons in nervous tissue [49]. Paclitaxel treatment is also shown to disrupt microtubule transport along axons thereby impairing protein and organelle trafficking within neurons [50, 51]. In particular, paclitaxel’s actions appear to correlate with deficits in mitochondrial trafficking in axons both *in vitro* as well as *in vivo* [50, 51] A schematic model of our proteomic findings on DRG function is presented in **Fig 5**. In this model, sensory deficits are suggested to arise as a function of protein changes within multiple cell types and processes in the DRG. In addition, paclitaxel associated proteomic changes may impact sensory signaling outside of the DRG through interactions of sensory axons with target tissue (e.g. epidermis) thereby contributing to pain sensitivity.

Our model is consistent with evidence on the involvement of DRG connectivity in chemotherapy associated neuropathy [52]. The DRG contains various sensory neurons, which serve in the processing of sensory information between periphery and CNS. These sensory neurons are encapsulated by a satellite glial cell (SGC) network that modulates neuronal responses to nociception [53]. In addition, immune cells can enter the DRG under heightened inflammatory conditions [54]. Our findings support the involvement of neurons and glia in the effect of paclitaxel within the DRG [17] as evidenced by changes in the expression of multiple cell type proteins. For example, Pvalb a calcium binding protein that identifies a subpopulation of proprioceptive DRG neurons and has been shown to be associated with peripheral nerve injury [55], is found to be significantly decreased by paclitaxel. S100b on the other hand, a calcium binding protein expressed in glial cells of the DRG [56], is also found to be decreased within the paclitaxel treatment group. These findings point to an effect of paclitaxel on various cell types within the DRG and enables hypothesis to support future mechanistic studies of PIPN.

Bioinformatic analysis of paclitaxel associated proteomic changes within the DRG defines 11 functional network clusters with components that are already known to contribute to pain responses in animal models [57–59]. Our results show proteins across clusters that contribute to enhanced inflammatory responses. For example, changes in the expression of immune cell markers including orosomucoid 1 protein (Orm1), an acute phase inflammatory response protein, and CD47 support evidence on increased neuroimmune activity within the DRG during paclitaxel treatment [60]. Changes in immune cell activity may also contribute to a loss in oligodendrocyte function including myelin production during neuropathic pain [61]. In support of this, our results show that myelin basic protein (Mbp) is significantly altered within the DRG in response to paclitaxel.

Non-neuronal cells and particularly SGCs are increasingly recognized for their role in the etiology of neuropathic pain. SGC are coupled via gap junctions that are modified by nerve injury as well as neuroinflammation [54]. Chemotherapeutic drugs, such as oxaliplatin or paclitaxel, have been shown to alter the number of gap junctions between SGCs leading to increased electrical coupling and calcium oscillatory activity within the DRG [62, 63]. In addition, SGC transmit signals bidirectionally between neuron and glia through purinergic (ATP) signaling [64]. Our data shows that Cluster 7 is enriched in purine metabolism proteins that are important for ATP signaling. Changes in ATP sensitivity within the DRG have been previously demonstrated in a mouse pain model with mechanical hypersensitivity and DRG sensory neuron hyperexcitability [45, 65, 66]. An analysis of differentially expressed proteins points to an enrichment of ATP regulators that are associated with mitochondrial activity. The largest of our clusters (Cluster 1) represents mitochondrial proteins including cytochrome C oxidase subunit 7c (Cox7c), mitochondrial encoded cytochrome C oxidase 1 (mt-Co1), thioredoxin 2 (Txn2), and hypoxia-inducible domain family member 1a (Higd1a). Mitochondrial dysfunction is a common feature of neuropathic pain including PIPN [42]. Changes in mitochondrial proteins can impact multiple cell types within the DRG and may influence processes such as schwann cell myelination [67].

Our findings involve nitrogen metabolism (Cluster 6), which fits in with existing research on nitrogen-related compounds in neuropathic pain. Namely, myoglobin (Mb) has been shown to be involved in pain processes [68]. Within this pathway, nitric oxide (NO) is important in a number of signaling pathways, including immune regulation, neuronal survival, and synaptic plasticity [69], and an overproduction of NO is associated with neuropathic pain [70]. Molecules associated with nitrogen metabolism have been shown to modulate sensory neuron excitability and nociceptive effects [71]. Within Cluster 6, we find that the fatty acid binding protein 4 (FABP4) is significantly increased within the DRG in response to paclitaxel. FABP4 is expressed in adipocytes as well as immune cells such as macrophages and participates in lipid signaling as well as nitrogen metabolism. Studies show that FABP4 can contribute to neuroinflammation [72].

Our findings provide insight on mechanisms that can contribute to pain responses following paclitaxel treatment within our PIPN model. Our proteomic results are consistent with earlier findings using this mouse model showing an effect of paclitaxel on mechanical and thermal sensitivity. This result is also similar to proteomic studies of paclitaxel associated neuropathy within the rat DRG [41]. Understanding the mechanisms that contribute to PIPN is an important research direction that can be leveraged through combining several OMIC technologies including RNA sequencing (RNAseq) and proteomics withing these important preclinical models.

## Supporting information

S1 File (DOCX)
