# Supplementary material for: Proteomic analysis of dorsal root ganglia in a mouse model of paclitaxel-induced neuropathic pain
Source: PLoS One. 2024 Sep 27;19(9):e0306498. doi: 10.1371/journal.pone.0306498 (PMC11432834; doi:10.1371/journal.pone.0306498)
Supplement: S1 File — (DOCX) [file pone.0306498.s001.docx]

**Supplement 1**

Supplement Table 1: Total number of proteins identified in cytosolic and membrane fractions

Supplement Table 2: Membrane proteins significantly downregulated by paclitaxel treatment

Supplement Table 3: Membrane proteins significantly upregulated by paclitaxel treatment

Supplement Table 4: Cytosolic proteins significantly downregulated by paclitaxel treatment

Supplement Table 5: Cytosolic proteins significantly upregulated by paclitaxel treatment

Supplement Table 6: DRG proteins shared with another pain mouse model

Supplement Figure 1. Venn diagram showing common DRG proteins between our dataset and other mouse models of neuropathy.

*Supplement Table 1:* Total number of proteins identified in cytosolic and membrane fractions

| **Fraction** | **Total identified proteins** | **Significantly altered proteins** |
| --- | --- | --- |
| Cytosolic | 2055 | 63 |
| Membrane | 2676 | 102 |

*Supplement Table 2: Membrane proteins significantly downregulated by paclitaxel treatment*

| **Gene Symbol** | **Description** | **Fold change** | **Adj. p-value** |
| --- | --- | --- | --- |
| Cd9 | CD9 antigen | 7.111061015 | 0.0000000774 |
| Apoh | Beta-2-glycoprotein 1 | 1.580296777 | 0.026284712 |
| S100b | Protein S100-B | 15.75067199 | 0.000000000000000178 |
| Cds2 | phosphatidate cytidylyltransferase 2 | 1.557305175 | 0.02771372 |
| Clgn | calmegin | 1.477216745 | 0.033326005 |
| Gnb4 | Guanine nucleotide-binding protein subunit beta-4 | 3.91458454 | 0.000121735 |
| Ndufs4 | NADH dehydrogenase [ubiquinone] iron-sulfur protein 4, mitochondrial | 1.523209702 | 0.029977147 |
| Gorasp2 | Golgi reassembly-stacking protein 2 | 1.764414341 | 0.017202266 |
| Mgst3 | Microsomal glutathione S-transferase 3 | 1.387491012 | 0.040974059 |
| Acot2 | Acyl-coenzyme A thioesterase 2, mitochondrial | 2.02884397 | 0.009357418 |
| Rtn3 | Isoform 3 of Reticulon-3 | 1.722030185 | 0.018965741 |
| Rps7 | 40S ribosomal protein S7 | 1.52292555 | 0.029996767 |
| Tlcd3b | Protein FAM57B | 1.614738564 | 0.024280713 |
| Rplp1 | 60S acidic ribosomal protein P1 | 3.54621092 | 0.000284308 |
| Pea15 | Astrocytic phosphoprotein PEA-15 | 2.547203586 | 0.002836589 |
| Stoml2 | Stomatin-like protein 2, mitochondrial | 1.92312136 | 0.011936545 |
| Esyt2 | Extended synaptotagmin-2 | 1.57966851 | 0.026322764 |
| Surf4 | surfeit locus protein 4 | 1.316045011 | 0.048300874 |
| Cd99 | CD99 antigen | 4.667442378 | 0.0000215 |
| Nap1l4 | Nucleosome assembly protein 1-like 4 | 1.941016736 | 0.011454688 |
| Zmpste24 | caax prenyl protease 1 homolog | 2.663843377 | 0.002168486 |
| Clic4 | Chloride intracellular channel protein 4 | 1.425880713 | 0.037507601 |
| Mtnd5 | NADH-ubiquinone oxidoreductase chain 5 | 2.657834419 | 0.002198698 |
| Mtdh | protein LYRIC | 1.530825698 | 0.029456036 |
| Mtco1 | Cytochrome c oxidase subunit 1 | 3.980892021 | 0.000104498 |
| Cd47 | Leukocyte surface antigen CD47 | 6.286245768 | 0.000000517 |
| Erlin1 | erlin-1 | 1.377042467 | 0.041971794 |
| Txn2 | Thioredoxin, mitochondrial | 2.928221058 | 0.00117972 |
| Pon2 | Serum paraoxonase/arylesterase 2 | 2.120163125 | 0.007582927 |
| Mpdu1 | Mannose-P-dolichol utilization defect 1 protein | 1.32074112 | 0.047781401 |
| Dpy30 | Protein dpy-30 homolog | 1.802289276 | 0.015765608 |
| Rap2b | ras-related protein Rap-2b | 2.36574719 | 0.004307773 |
| Dad1 | Dolichyl-diphosphooligosaccharide--protein glycosyltransferase subunit DAD1 | 6.528944574 | 0.000000296 |
| Dync1li2 | Cytoplasmic dynein 1 light intermediate chain 2 | 8.828958429 | 0.00000000148 |
| Golt1b | vesicle transport protein GOT1B | 2.411201855 | 0.0038797 |
| Stim1 | stromal interaction molecule 1 | 1.939668443 | 0.011490305 |
| Lgals3bp | Galectin-3-binding protein | 2.453761696 | 0.003517534 |
| Cers2 | Ceramide synthase 2 | 1.330626423 | 0.046706097 |
| Cds1 | Phosphatidate cytidylyltransferase 1 | 2.019352123 | 0.009564183 |
| Pmpcb | mitochondrial-processing peptidase subunit beta | 1.927714654 | 0.011810964 |
| Lyz2 | lysozyme c-2 | 1.944108846 | 0.011373422 |
| Mtatp8 | ATP synthase protein 8 | 9.990039469 | 0.000000000102 |
| Ppif | Peptidyl-prolyl cis-trans isomerase F, mitochondrial | 2.894496968 | 0.001274979 |
| Sigmar1 | sigma non-opioid intracellular receptor 1 | 1.897120428 | 0.012673004 |
| U2af2 | Splicing factor U2AF 65 kDa subunit | 1.446446276 | 0.035772865 |
| Ndufa3 | NADH dehydrogenase [ubiquinone] 1 alpha subcomplex subunit 3 | 5.435242826 | 0.00000367 |
| Derl1 | Derlin-1 | 2.631069497 | 0.002338463 |
| Kdelr1 | ER lumen protein-retaining receptor 1 | 3.644043023 | 0.000226964 |
| Tbc1d17 | TBC1 domain family member 17 | 1.314195208 | 0.048507042 |
| Gmpr | GMP reductase 1 | 2.030655575 | 0.009318466 |
| Slc1a4 | neutral amino acid transporter A | 2.447236663 | 0.003570782 |
| Tomm5 | mitochondrial import receptor subunit TOM5 homolog | 2.663843377 | 0.002168486 |
| Metap1 | Methionine aminopeptidase 1 | 3.724737031 | 0.000188479 |
| Entpd1 | Ectonucleoside triphosphate diphosphohydrolase 1 | 1.814572199 | 0.015325964 |
| Mfsd10 | Major facilitator superfamily domain-containing protein 10 | 1.620858493 | 0.023940957 |
| Bcl2l1 | bcl-2-like protein 1 | 2.557673437 | 0.002769023 |
| Orm1 | Alpha-1-acid glycoprotein 1 | 1.80457551 | 0.015682832 |
| Slc36a1 | Proton-coupled amino acid transporter 1 | 2.577737728 | 0.002644005 |
| Higd1a | HIG1 domain family member 1A, mitochondrial | 2.043808702 | 0.009040476 |
| Chd6 | Chromodomain-helicase-DNA-binding protein 6 | 2.393428082 | 0.004041773 |
| Mia2 | Melanoma inhibitory activity protein 2 | 1.997991177 | 0.010046362 |
| Cox7c | Cytochrome c oxidase subunit 7C, mitochondrial | 1.682980643 | 0.02075006 |
| Slc66a3 | PQ-loop repeat-containing protein 3 | 2.217873588 | 0.006055171 |
| Eif3m | Eukaryotic translation initiation factor 3 subunit M | 2.411201855 | 0.0038797 |
| Tmem167a | Protein kish-A | 2.928221058 | 0.00117972 |
| Tspan18 | Tetraspanin-18 | 8.313746364 | 0.00000000486E |
| Tmem259 | Membralin | 1.55642276 | 0.027770087 |
| Tmem256 | Transmembrane protein 256 | 1.732632156 | 0.018508356 |
| Tap1 | Antigen peptide transporter 1 | 1.603076857 | 0.024941533 |
| Thap11 | THAP domain-containing protein 11 | 2.003339809 | 0.009923393 |
| Icam2 | Intercellular adhesion molecule 2 | 8.084303173 | 0.00000000824 |
| Tmem186 | Transmembrane protein 186 | 3.752657778 | 0.000176743 |
| Ndst3 | Bifunctional heparan sulfate N-deacetylase/N-sulfotransferase 3 | 2.663843377 | 0.002168486 |
| Ptdss2 | phosphatidylserine synthase 2 | 1.313278283 | 0.048609563 |

*Supplement Table 3: Membrane proteins significantly upregulated by paclitaxel treatment*

| **Gene Symbol** | **Description** | **Fold change log_2_** | **Adj. p-value** |
| --- | --- | --- | --- |
| Anxa6 | annexin A6 | 1.579600507 | 0.026326886 |
| Mbp | Isoform 4 of Myelin basic protein | 1.572940881 | 0.026733703 |
| Pmp2 | Myelin P2 protein | 1.75756952 | 0.017475535 |
| Atp2a1 | Sarcoplasmic/endoplasmic reticulum calcium ATPase 1 | 1.530539777 | 0.029475435 |
| Calm2 | Calmodulin | 3.134705286 | 0.000733322 |
| S100a10 | Protein S100-A10 | 3.474400687 | 0.000335428 |
| S100a9 | Protein S100-A9 | 1.732632156 | 0.018508356 |
| Slc25a31 | ADP/ATP translocase 4 | 13.29211741 | 0.000000000000051 |
| Fabp4 | Fatty acid-binding protein, adipocyte | 2.663843377 | 0.002168486 |
| Pvalb | Parvalbumin alpha | 15.75067199 | 0.000000000000000178 |
| S100a6 | protein S100-A6 | 1.603076857 | 0.024941533 |
| Dynll1 | Dynein light chain 1, cytoplasmic | 1.342434418 | 0.045453317 |
| Prps1 | ribose-phosphate pyrophosphokinase 1 | 1.897120428 | 0.012673004 |
| Cep162 | Centrosomal protein of 162 kDa | 2.251158696 | 0.00560843 |
| Dpm1 | Dolichol-phosphate mannosyltransferase subunit 1 | 12.67548914 | 0.000000000000211 |
| Man2b1 | Lysosomal alpha-mannosidase | 1.447124175 | 0.03571707 |
| Cpsf6 | Cleavage and polyadenylation specificity factor subunit 6 | 1.414018689 | 0.038546177 |
| Inmt | Indolethylamine N-methyltransferase | 2.57010341 | 0.002690894 |
| Hmgn2 | Non-histone chromosomal protein HMG-17 | 3.10324402 | 0.000788417 |
| Myh10 | Myosin-10 | 1.436115884 | 0.036633981 |
| Mme | Neprilysin | 3.203742137 | 0.000625544 |
| Septin4 | septin-4 | 5.69086828 | 0.00000204 |
| Asap2 | Arf-GAP with SH3 domain, ANK repeat and PH domain-containing protein 2 | 1.810719726 | 0.01546252 |
| Fnbp1l | Formin-binding protein 1-like | 1.427525517 | 0.037365817 |
| Gucy2g | Guanylate cyclase 2G | 4.715230987 | 0.0000193 |
| Gng3 | Guanine nucleotide-binding protein G(I)/G(S)/G(O) subunit gamma-3 | 1.46000858 | 0.034673 |
| Rfx1 | MHC class II regulatory factor RFX1 | 1.680941539 | 0.020847715 |
| Sdk1 | Protein sidekick-1 | 2.239830703 | 0.005756643 |

*Supplement Table 4: Cytosolic proteins significantly downregulated by paclitaxel treatment*

| **Gene Symbol** | **Description** | **Fold change log_2_** | **Adj. p-value** |
| --- | --- | --- | --- |
| Mbp | Isoform 11 of Myelin basic protein | 16.26121944 | 0.0000000000000000548 |
| Naca | Nascent polypeptide-associated complex subunit alpha, muscle-specific form | 1.327174615 | 0.0470788 |
| Tpm3 | Isoform 2 of Tropomyosin alpha-3 chain | 2.645200507 | 0.002263599 |
| Ppp1ca | serine/threonine-protein phosphatase PP1-alpha catalytic subunit | 1.324773728 | 0.047339784 |
| Mlec | Malectin | 2.145853614 | 0.007147372 |
| Cltb | clathrin light chain B | 2.519014553 | 0.003026812 |
| Pvalb | Parvalbumin alpha | 13.32422166 | 0.0000000000000474 |
| Atp5f1d | ATP synthase subunit delta, mitochondrial | 1.403372329 | 0.039502781 |
| Btf3l4 | transcription factor BTF3 homolog 4 | 1.8926909 | 0.012802922 |
| Rps28 | 40S ribosomal protein S28 | 1.892188881 | 0.01281773 |
| Naa15 | N-alpha-acetyltransferase 15, NatA auxiliary subunit | 1.554831586 | 0.027872018 |
| Tbca | Tubulin-specific chaperone A | 1.894530624 | 0.012748802 |
| Hsd17b8 | estradiol 17-beta-dehydrogenase 8 | 1.405154462 | 0.039341013 |
| Cst3 | Cystatin-C | 2.342944434 | 0.004539997 |
| Emc4 | ER membrane protein complex subunit 4 | 5.354577731 | 0.00000442 |
| Rps21 | 40S ribosomal protein S21 | 4.818156412 | 0.0000152 |
| Atp5f1e | ATP synthase subunit epsilon, mitochondrial | 1.783038576 | 0.01648016 |
| Hebp2 | Heme-binding protein 2 | 2.191294429 | 0.006437327 |
| Stk24 | Serine/threonine-protein kinase 24 | 1.356872465 | 0.043967071 |
| Lyrm4 | LYR motif-containing protein 4 | 2.012704786 | 0.009711699 |
| Adam23 | Disintegrin and metalloproteinase domain-containing protein 23 | 1.583754631 | 0.026076264 |
| Tmod2 | Tropomodulin-2 | 3.902805183 | 0.000125082 |
| Cdc42bpb | Serine/threonine-protein kinase MRCK beta | 1.328898336 | 0.046892314 |
| Arhgap22 | Rho GTPase-activating protein 22 | 11.29328222 | 0.00000000000509 |
| Ptgds | Prostaglandin-H2 D-isomerase | 1.337744862 | 0.045946786 |
| Clpp | ATP-dependent Clp protease proteolytic subunit, mitochondrial | 3.329328413 | 0.000468459 |
| Slmap | Sarcolemmal membrane-associated protein | 1.913375686 | 0.012207432 |

*Supplement Table 5: Cytosolic proteins significantly upregulated by paclitaxel treatment*

| **Gene Symbol** | **Description** | **Fold change log_2_** | **Adj. p-value** |
| --- | --- | --- | --- |
| Ywhae | 14-3-3 protein epsilon | 1.331967422 | 0.046562102 |
| Serpinb6 | serpin B6 | 1.616799057 | 0.024165787 |
| Dbi | acyl-CoA-binding protein | 1.754640201 | 0.017593806 |
| Cd9 | CD9 antigen | 1.80090274 | 0.015816022 |
| Entpd2 | ectonucleoside triphosphate diphosphohydrolase 2 | 3.192070768 | 0.000642583 |
| Ca2 | Carbonic anhydrase 2 | 2.24226282 | 0.005724495 |
| S100b | Protein S100-B | 2.094451711 | 0.008045412 |
| Pgm1 | Phosphoglucomutase-1 | 2.754807002 | 0.001758705 |
| Ca3 | carbonic anhydrase 3 | 1.707299115 | 0.019620085 |
| Calm2 | Calmodulin | 3.875583817 | 0.000133173 |
| Vapa | vesicle-associated membrane protein-associated protein A | 2.026009405 | 0.009418692 |
| Mtpn | Myotrophin | 1.830638647 | 0.014769349 |
| Get3 | ATPase ASNA1 | 1.590937792 | 0.025648514 |
| Apoo | MICOS complex subunit MIC26 | 1.797923941 | 0.015924876 |
| Gpd1 | Glycerol-3-phosphate dehydrogenase [NAD(+)], cytoplasmic | 3.648772048 | 0.000224506 |
| Ttr | Transthyretin | 1.80090274 | 0.015816022 |
| Mb | Myoglobin | 3.250638638 | 0.000561515 |
| Psmd3 | 26S proteasome non-ATPase regulatory subunit 3 | 1.529262015 | 0.029562284 |
| Psma4 | Proteasome subunit alpha type-4 | 2.048161825 | 0.008950312 |
| Reep5 | Receptor expression-enhancing protein 5 | 2.933643135 | 0.001165083 |
| G6pdx | Glucose-6-phosphate 1-dehydrogenase X | 1.711047639 | 0.019451467 |
| Cntf | Ciliary neurotrophic factor | 3.037745292 | 0.000916758 |
| Mfn2 | Mitofusin-2 | 3.964626303 | 0.000108486 |
| Tagln | transgelin | 1.679782342 | 0.020903435 |
| Gnb3 | Guanine nucleotide-binding protein G(I)/G(S)/G(T) subunit beta-3 | 2.866897202 | 0.001358635 |
| Gspt1 | Eukaryotic peptide chain release factor GTP-binding subunit ERF3A | 4.116906641 | 0.0000764 |
| Gpd1l | Glycerol-3-phosphate dehydrogenase 1-like protein | 2.284724124 | 0.005191297 |
| Eif4e | Eukaryotic translation initiation factor 4E | 1.48875231 | 0.032452465 |
| Tsn | Translin | 1.405018579 | 0.039353324 |
| Carhsp1 | Calcium-regulated heat stable protein 1 | 2.342643975 | 0.004543139 |
| Kif27 | kinesin-like protein KIF27 | 1.910273339 | 0.012294947 |
| Ppef2 | Serine/threonine-protein phosphatase with EF-hands 2 | 1.998619966 | 0.010031827 |
| Gpat4 | glycerol-3-phosphate acyltransferase 4 | 2.996402049 | 0.001008319 |
| Apc2 | adenomatous polyposis coli protein 2 | 1.975581945 | 0.010578353 |
| Ces3b | Carboxylesterase 3B | 5.785156152 | 0.00000164 |
| Sdk1 | Protein sidekick-1 | 1.478180948 | 0.033252098 |

*Supplement Table 6:* DRG proteins shared with another pain mouse model.

| Ttc38 | Pld3 | Nono | Rbbp4 | Atp1b1 | Hdgf | Cavin1 | Armc1 | Dazap1 | Mfn2 |
| --- | --- | --- | --- | --- | --- | --- | --- | --- | --- |
| Nsdhl | Cbr3 | Eif3g | Ndrg3 | Sez6l2 | Tmx1 | Drg2 | Arhgdia | Zc3h15 | Eps15 |
| Acad9 | Rnpep | Psmd2 | Isoc2a | Msn | Tsc22d1 | Tmed2 | Coro1a | Comt | Pcca |
| Dcps | Ptpa | Hnrnpul1 | Rap1b | Ezr | Acot9 | Arpc1a | Psma3 | Zyx | Abca2 |
| Gde1 | Arsa | Fxyd7 | Psmd6 | Rdx | Hspa4 | Ptges3 | Itm2b | Trnt1 | Pds5a |
| Eps15l1 | Tmod2 | Mapre2 | Lars2 | Psmd14 | Ncln | Reep5 | Wbp2 | Sec22b | Emc10 |
| Fgf1 | Sts | Ufm1 | Ndrg2 | Jam3 | Fgg | Prkar2b | Psmd4 | Dpysl2 | Tbc1d17 |
| Haghl | Plec | Hadh | Clgn | Ccdc47 | Capza1 | Mpdu1 | Taco1 | Hspa4l | Aco1 |
| Mpi | Hspe1 | Hnrnpk | Cnn3 | Mpc2 | Mospd2 | Lamp2 | Stx7 | Clpp | Serpinh1 |
| Picalm | Ccdc124 | Adpgk | Pepd | Ndufa11 | Capza2 | Aldoc | S100a8 | Apmap | Czib |
| Hmgcs2 | Impa1 | Sfxn1 | Ndufv3 | Chmp4b | Ubl4a | Ddost | Aoc3 | Pafah1b3 | Prrc2a |
| Abi2 | Pgrmc1 | Cpe | Psmc4 | Prelp | Ap3m1 | Aldoa | Nap1l4 | Gpc6 | Ppp3r1 |
| Kcnab2 | Dbn1 | Ppp2r1a | Pals2 | Tln1 | Cend1 | Fetub | Apex1 | Pafah1b2 | Hsd17b8 |
| Hnrnpdl | Scn4b | Scg2 | Kdsr | Hnrnpab | Ak1 | Fbxo2 | Psmg2 | Ado | Fabp3 |
| Eri3 | Arsb | Tollip | Ndufs8 | Niban1 | Cnih4 | Sdha | Anp32b | Psap | Map6 |
| Atp5po | Entpd2 | Nagk | Rpl7a | S100a6 | Agfg1 | Tubb2b | Ahcy | Cox5a | Purb |
| Fads2 | Copb2 | Nutf2 | Ufsp2 | Sh3glb1 | Cd81 | Gnpda1 | Dcakd | Eef1a1 | Map2 |
| Nes | Dgke | Rpl11 | Rimkla | Rpl26 | Nid1 | Iars2 | Ddt | Ubxn1 | Ganab |
| Aldh3b1 | Txnl1 | Cacybp | Nit1 | Pdk2 | Copg2 | Col12a1 | Cnpy4 | Psmc3 | Cox20 |
| Cab39l | Hnrnpa0 | Rab1b | Jup | Vps26a | Atg7 | Tgm2 | Gnai2 | Rab4a | Dnajc6 |
| Itih5 | Psmd12 | Adam11 | Ggt7 | Blvrb | Gpx3 | Lin7c | Macrod1 | Atp5mk | Fsd1l |
| Mrpl12 | Npc2 | Hadhb | Cers2 | Atp6v0d1 | Aldh3a2 | Hspb8 | Echs1 | Acyp1 | Dhx15 |
| Actr2 | Fam20b | Adam22 | Pdia4 | U2af1 | Idh2 | Acadm | Ttc1 | Tbl2 | Aqp1 |
| Myo5a | Rtn4ip1 | Tst | Hdlbp | Arfgef2 | Ist1 | Pdhb | Fam162a | Acyp2 | Stt3a |
| Hyou1 | Mrps30 | Dld | Ube2m | Timm50 | Tpm3 | Rpl4 | Cpt1a | Lgals9 | Slc9a9 |
| Hmgcs1 | Cnpy2 | Lman2 | Ctnnb1 | Ncam1 | Aldh2 | Sco1 | Ppp2r5e | Atp5f1e | Commd3 |
| Rab11a | Pfas | Adam23 | Sumo3 | Septin7 | Kif21a | Myg1 | Timm29 | Mapk3 | P4hb |
| Actr1a | Cryzl2 | Ndufa12 | Tomm40 | Acot1 | Dffa | Npepps | Cdr2l | Lamp1 | Fnbp1 |
| Bpnt1 | Lman1 | Aldh9a1 | Timm17a | Eif6 | Ctnna2 | Emb | Dars1 | Cda | Psmf1 |
| Letmd1 | Slc2a1 | Uba5 | Psma4 | Map7d2 | Glb1 | Otud6b | Tmx3 | Anxa1 | Cep170 |
| Cpne6 | Pgm1 | Cd55 | L1cam | Pnpt1 | Eef1g | Syn1 | Nckipsd | Dynlt3 | Psmc1 |
| Stoml2 | Brox | Plpp3 | Appl1 | Myl1 | Fntb | Glrx2 | Ptbp2 | Naca | Ube3c |
| Rabac1 | Sec11c | Actr3 | Aimp1 | Golga1 | Fmo2 | Dhcr24 | Twf1 | Tubb4b | Stxbp3 |
| Slc43a3 | Abhd14b | Tpp2 | Psmb3 | Naaa | Anxa6 | Snx2 | Tpp1 | Emd | Hectd3 |
| Napa | Cox7a2l | Gorasp2 | Psma1 | Ece1 | Wdr77 | Bax | Afm | Tuba1a | Sarm1 |
| Plbd2 | Man2b1 | Gk | Psmb2 | Rpl38 | Pfdn1 | Prdx4 | Praf2 | Lclat1 | Htra2 |
| Calca | Rsu1 | Sdhd | Fkbp2 | Surf4 | Cdc42 | Galk1 | Dglucy | Alyref | Nipa1 |
| Acsl5 | Mme | Tax1bp3 | Ube2n | S100a11 | Gatm | Atic | Rpn1 | Tuba4a | Prps1 |
| Pcsk1n | Hnrnpm | Eif3m | Ube2k | Cd34 | Snx5 | Cavin2 | Pdha1 | Clta | Immt |
| Vapb | Cyc1 | Slc1a3 | Clic4 | Acadvl | Mdp1 | Elavl2 | Mapre1 | Mlip | Faf1 |
| Pde6d | Prpsap1 | Naa50 | Gnl1 | Rpl35a | Psmc2 | Prdx1 | Lman2l | Cyb5a | Acaa1a |
| Itih4 | Dynll2 | Ap2b1 | Add3 | Atp2a2 | Uqcc1 | Hsp90b1 | Mars1 | Apool | Btf3 |
| Cd9 | Ntrk2 | Psmd5 | Add2 | Pex14 | Tmem143 | Abcf1 | Ppp1r7 | Cox7a1 | Chp1 |
| Nudt5 | Xpo1 | Hsd17b10 | Odr4 | Lamc1 | Map1b | Tubb1 | Cnrip1 | Cox6b1 | Ass1 |
| Cyb5r1 | Hmbox1 | Srpra | Cntn1 | Lamb2 | Jagn1 | Far1 | Prdx2 | Tomm5 | Rab5b |
| Sod3 | Pex5l | Rpn2 | Tagln3 | Mob4 | Vps4b | Snx3 | Ahcyl2 | Sntb2 | Rab8b |
| Uqcrc2 | Gstt1 | Plin3 | Aifm1 | Arfip2 | Spcs1 | Tln2 | Lgmn | Fnta | Rab10 |
| Trio | Ywhag | Gspt1 | Sec23ip | Slc25a5 | Matr3 | Snx12 | Pnp | Cox7a2 | Clptm1l |
| Lipa | Rnmt | Alcam | Tmem11 | Fabp7 | Slirp | Esyt2 | Cdv3 | Lcp1 | Etfdh |
| Cmpk2 | Armc10 | Dnajc7 | Slc22a18 | Lyrm4 | Psmd8 | Cldn19 | Ano10 | Snta1 | Sorbs2 |
| Afg3l2 | Micu1 | Tbcb | Add1 | Fkbp1a | Nsf | Tm9sf3 | Mrrf | Mcu | Tom1l2 |
| Ddx6 | Gbp2 | Lims1 | Opa3 | Rab4b | Gsr | Synm | Copb1 | Dnm3 | Tardbp |
| Adsl | H2bc3 | Ptgr2 | Acox1 | Cdc42bpb | Ttc9 | Rps8 | B3gat3 | Lamtor3 | Gdi1 |
| Eif3b | Ncdn | Pitrm1 | Coq9 | Kpnb1 | Bola1 | Rps15a | Gstt2 | Deptor | Anxa5 |
| Serpinb1a | Sptan1 | Psat1 | Bpnt2 | Lum | Pcna | Hsdl2 | Actr1b | Dnajb4 | Hnrnpll |
| Tmed5 | Tpm4 | Mrps23 | Stx1b | Tmed3 | Chm | Tns3 | Vat1 | Serpinf2 | Pfdn2 |
| Gclm | Cltb | Copz1 | Serpina1a | Pcp4l1 | Acss2 | Zdhhc17 | Ckap5 | Dnm1l | Asrgl1 |
| Cnpy3 | Oxct1 | Blvra | Serpina3k | Naga | Ddah1 | Phldb1 | Psmd7 | Hsp90ab1 | Glul |
| Amacr | Acsf2 | Ppp1r21 | Ctnnd1 | Septin9 | Nup93 | Pdxk | Cpd | Paqr4 | Micu3 |
| Prxl2b | Mrc2 | Sirt2 | Tceal5 | Mia3 | Wfs1 | Poglut1 | Cp | Pdpr | Nherf2 |
| Arl6ip1 | Ppm1a | Aldh7a1 | Cd44 | Uqcrb | Rexo2 | Tbcd | Atl1 | Cfh | Hax1 |
| Cd82 | Inpp1 | Dnaja2 | Lyrm7 | Smpdl3a | Arfgap3 | Pdk3 | Idh3a | Drp2 | Basp1 |
| Hint2 | Sncg | Serbp1 | Adam10 | Adprh | Iqgap1 | Dpp7 | F13a1 | Cbr1 | Cask |
| Eif2s3x | Sord | Pmpcb | Tgfbi | Arl1 | Eml1 | Lpgat1 | Ehd2 | Msrb2 | Eif1 |
| Slc25a13 | Lama4 | Cluh | Clptm1 | Nipsnap2 | Nhlrc2 | Ywhae | Itga3 | Scn11a | Rab31 |
| Snca | Hdac1 | Trim36 | Fam98a | Kif5a | Tmtc3 | Rbm3 | Gopc | Ppp1r11 | Ciapin1 |
| Fkbp8 | Nploc4 | Abat | Arcn1 | Kif1a | Nlrx1 | Fmo1 | Ttll12 | Capn2 | Hepacam |
| Gpr89 | Ptms | Tmed10 | Mug1 | Nipsnap1 | Tstd3 | Arl6ip5 | Slc44a2 | Galnt17 | Maoa |
| Adrm1 | Napg | Ssr1 | Lrrc47 | Ufl1 | Actb | Sdf2l1 | Ric8a | Cacna2d1 | Cog5 |
| Lmf2 | Lyz2 | Alg2 | Alb | Lrp1 | Cct4 | Ppm1e | Gstm5 | Iscu | Anp32a |
| Psip1 | Arl2 | Eif2a | Napb | Pcyt1a | Cct2 | Rps18 | Gbe1 | Ppa1 | Cdh2 |
| Mvd | Phpt1 | Rab8a | Nckap1 | Ermp1 | Mrpl38 | Cox7c | Them4 | Gyg1 | Tm7sf2 |
| Lsm12 | Rbm8a | Mtmr6 | Septin4 | Nae1 | Chdh | Rps14 | Usp5 | Slc12a2 | Oat |
| Vdac2 | Cc2d1b | Rragc | Pabpn1 | Bpgm | Cct7 | Ttl | Tubb6 | Bcam | Pip4k2c |
| Vdac3 | Calb2 | Lgi3 | Psme3 | Prph | Scfd1 | Mmut | Dmxl2 | Bin1 | Hsd17b4 |
| Vdac1 | Uggt1 | Csad | Sncb | Mogs | Sugt1 | Rps23 | Rtn1 | Eloc | Cap1 |
| Ndufaf2 | Ptgds | Ccar2 | Colgalt1 | Naa15 | Islr | Cnp | Map2k1 | Cds1 | Cpt1c |
| Zmpste24 | Ndufb11 | Mical1 | Atxn10 | Tigar | Rplp0 | Phf24 | Nucb1 | Prune2 | Lsm3 |
| Ehd3 | Tdrkh | Cdipt | Nap1l1 | Bgn | Tbcc | Hspa12a | Rrbp1 | Abhd10 | Ide |
| Tars1 | Rars1 | Vps28 | Bcl2l1 | Dcn | Derl1 | Cope | Usp15 | Lmf1 | Fbln1 |
| Rhot2 | Mrps27 | Prdx6 | Rab6b | Cops8 | Gcat | Dnajc3 | Trim2 | Usp8 | Snrpd2 |
| Gng12 | Adk | Ctsd | Atp2b2 | Ctsa | Stx8 | Ahsg | Vbp1 | Pdp1 | Tmco1 |
| Ech1 | Dctn1 | Tapbp | Rbm14 | Syngr1 | Cct3 | Pgap1 | Pcyt2 | Psmc5 | Rbp1 |
| Clcn6 | Prkcsh | Dctn3 | Rimoc1 | Cct8 | Cct6a | Acsbg1 | Urod | Khdrbs1 | Snrpd1 |
| Coq8a | Cyfip1 | Rbbp9 | Lgals3bp | Prx | Fam98b | Vwa8 | Gaa | Anxa2 | Nudt7 |
| Aldh18a1 | Slc25a4 | Abcb8 | Cd151 | Strn | Cct5 | Dync1li2 | Ctps1 | Pdia6 | Enoph1 |
| Zfpl1 | Pls3 | Slc25a1 | Abce1 | Bcat1 | Lsm4 | Hmgb2 | Arf4 | Chid1 | Cox5b |
| Abhd4 | Prkar1a | Tspan15 | Pgrmc2 | Sbds | Lrpap1 | Abi1 | Cadps | Clasp1 | Ptgr3 |
| Anxa7 | Ahsa1 | Idh1 | Rap2b | Sars2 | Ncl | Tmem165 | Diras1 | Chd4 | Snrpd3 |
| Pigu | Sgta | Kpna1 | Arhgap1 | Atp6v1a | Stip1 | Agk | Tmem65 | Apoc3 | Metap1 |
| Clip2 | Dhrs7 | Pla2g7 | Prkaca | Atp6v1e1 | Caprin1 | Col15a1 | Mthfd1 | Uba1 | Clybl |
| Ndufaf3 | Eif4g1 | Sae1 | Gnai1 | Ggct | Esd | Kcna1 | Abcd3 | Nedd4 | Ndufa13 |
| Tmem126a | Atp1a1 | Plekhb1 | Hbb-b1 | Arpc5l | Dstn | Pgs1 | Grhpr | Elovl5 | Capn1 |
| Celf2 | Aimp2 | Septin6 | Pa2g4 | Sqstm1 | Mapre3 | Abcb9 | Ndufv1 | Ralb | Pin1 |
| Trappc3 | Tecr | Papss1 | Dguok | Dst | Sec11a | Pura | Fam210a | Pycr2 | Prep |
| Naa10 | Blmh | Prrc2c | Cox4i1 | Isg15 | Uchl1 | Acad8 | Cpt2 | Gcdh | Arf6 |
| Nme2 | Cndp2 | Myh9 | Cd59a | Cat | Os9 | Arpc2 | Bckdhb | Mtarc2 | Sfpq |
| Folh1 | Rcc2 | Ywhah | Comtd1 | Gss | Reep2 | Snrpb | Akr1a1 | Lrrc59 | Kpna3 |
| Rps2 | Ncmap | Tkt | Tanc2 | Dusp3 | Hacd3 | Slc27a1 | Sf1 | Nedd8 | Rheb |
| Acp3 | Dhdh | Rdh11 | Sh3bgrl | Itpa | Pgk1 | Gnaz | Tsg101 | Dctn4 | Psmc6 |
| Psmb1 | Atl3 | Exog | Kank4 | Otub1 | Ehd1 | H2-Ab1 | Eif4h | Mrpl19 | Sgpl1 |
| Adh5 | Ndufv2 | Hnrnpd | Calu | Myl12b | Kbtbd11 | Sec61a1 | Slc12a9 | Eif3c | Cotl1 |
| Selenof | Ndufb8 | Rps11 | Uba3 | Ipo4 | Gabarapl1 | Gamt | Phb1 | Rpl7 | Gmfb |
| Rad23b | Fah | Pcbp2 | Maob | Lta4h | Stmn2 | Rangap1 | Cadm3 | Rps21 | C1qbp |
| Rad23a | Psmb6 | Gfm1 | Dpysl5 | Ighg1 | Emc8 | Mapk9 | H2-K1 | Fdft1 | Vta1 |
| Lnpep | Fgb | Get1 | Gnb1 | Klc2 | Efnb1 | Scn8a | Eif2s1 | Scrn1 | Tmem35a |
| Selenot | Fabp5 | Gmds | Rmnd1 | Cand1 | Surf1 | Hebp2 | Hadha | Aacs | Atp5pb |
| Sh3bgrl2 | Alpl | Pcmt1 | Agpat3 | Pmp2 | Art3 | Dlat | Coq3 | Mdh1 | Rpl27a |
| Nomo1 | Gabarapl2 | Rps29 | Gnb2 | Mtx2 | Bckdha | Pitpnm1 | Kyat3 | Ndufb10 | Ndufab1 |
| Hspa9 | Fkbp3 | Lama2 | Gnb4 | Atp5mf | Phb2 | Ybx1 | Acbd5 | Dlg1 | Itpr3 |
| Hars1 | Gfus | Ppp5c | Gnb5 | Nherf1 | Grcc10 | Rpl36a | Rpl10 | Mecr | Nceh1 |
| Pak3 | Thnsl1 | Dmd | Slc1a4 | Gstp1 | Pygm | Dync1i2 | Slc3a2 | Rab35 | Sdhc |
| Degs1 | Gpt2 | Snap47 | Nudt9 | Atxn2l | Csde1 | Tubg1 | Mthfd1l | Got1 | Tusc3 |
| Sec23a | Eif4g2 | Man1a2 | Rpl30 | Fxn | Dctn6 | Dync1i1 | Arl3 | Got2 | Scamp3 |
| H2ac25 | Vat1l | Dusp15 | Eif3e | H2-D1 | Acox3 | Pfn1 | Smap1 | Nptn | Ddah2 |
| Snrnp70 | Vamp1 | Cmtm5 | Ptpn11 | Atp6v1b2 | Cbr4 | Pdia3 | Cfb | Uqcc2 | Gps1 |
| App | Git1 | Ahcyl1 | Gstm2 | Tagln | Mb | Chchd6 | Rps27 | Magt1 | Npc1 |
| Psmd3 | Bri3bp | Fam234a | Rpl39 | Ran | Ipo7 | Glud1 | Sparc | Lss | Letm1 |
| Hk1 | Tiprl | Mff | Ctnna3 | Rbms3 | Slc44a1 | Pdcd6ip | Itih1 | Nudc | Ppid |
| Psma6 | Phyhipl | Gpd1l | Serpina1e | Creld1 | Elapor2 | Acsf3 | Itih2 | Ndufa1 | Suclg2 |
| Ptpmt1 | Slc25a12 | Rpl22 | Serpina1d | Ptges2 | Gpld1 | Eif5 | Itih3 | Ppm1f | Atp2c1 |
| Strn3 | Pir | Tubb4a | Myef2 | Apoh | Selenbp1 | Spryd7 | Coro1b | Apoo | Adprs |
| Lpcat4 | Coro2b | Tmem62 | Gna13 | Wnk1 | Scarb2 | Gphn | Coro1c | Uqcr10 | Ilk |
| Lima1 | Tmx2 | Ncstn | Avil | B2m | Ckap4 | Rhoc | Suclg1 | Ctsb | Rps16 |
| Tsn | Cfi | Mtch2 | Cycs | Pak2 | Ndufb6 | Slmap | Sorcs2 | Septin5 | Sucla2 |
| Rhot1 | Tbrg4 | Pigs | Pdlim5 | Gns | Prkg1 | Plgrkt | Dbi | Mpp1 | Apoa1 |
| Cntf | Bola2 | Nit2 | Aldh4a1 | Tufm | Brk1 | Slc25a24 | Rpl35 | Gmpr | Etfa |
| Vps26b | Canx | Cdh1 | Eif3h | Farsa | Map1lc3a | Ppa2 | Nefm | Stam | Ufc1 |
| Cbfb | Eef1d | Sgce | Spryd4 | Psmd11 | Ddx1 | Mrpl23 | Nefl | Pebp1 | Ndufa10 |
| Opa1 | Ephb6 | Mboat2 | Gcsh | Osbpl1a | Rp2 | Rbp4 | Nras | Csrp1 | Bet1 |
| Cpsf6 | Actn4 | Fabp4 | Trarg1 | Cpox | Ssr4 | Prkra | Kras | Rufy3 | Pmm1 |
| Sars1 | Alg11 | Xpnpep1 | Fsd1 | Tubb3 | Dpysl3 | Atp13a1 | Rpl28 | Myadm | G6pdx |
| Tmpo | Cept1 | Pgam2 | Plaa | Acaa2 | Ttn | Rps27a | Phgdh | Bsg | Kif5c |
| Inmt | Cstb | Stx12 | Steap3 | Sar1a | Lars1 | Rpl8 | Rpl13a | Sort1 | Mat2b |
| G3bp1 | Dad1 | Cfl1 | Vti1b | Apip | Eif1ax | Eci2 | Mif | Cyb5b | Naxe |
| Tfrc | C8b | Hmox2 | Nucb2 | Pptc7 | Stub1 | Arpc1b | Qdpr | Fbxo7 | Dhrs4 |
| Ap1m1 | Ndufa4 | Ap2a2 | Atp5me | Gatd1 | Ppp1ca | Lasp1 | Ccdc51 | Mrps36 | Trap1 |
| Itgb4 | Cltc | Ap2a1 | Mvp | Cspg4 | Atp5f1c | Acp2 | Mpv17 | Fam136a | Tmem14c |
| Asns | Sh3gl2 | Rala | Vasp | Fxr2 | Atp5if1 | Rpl32 | Gosr2 | Mgll | Chl1 |
| Fstl1 | Ostf1 | Rps12 | Them6 | Idh3g | Gnao1 | Rbm39 | Ap1g1 | Isoc1 | Col1a1 |
| Snrpe | Dysf | Por | Api5 | Pithd1 | Rps9 | Ptpn23 | Kif5b | Rpl27 | Dop1b |
| Snrpf | Wdr37 | Ppp3ca | Acta1 | Pdk1 | Ncam2 | Serpinc1 | Slc25a25 | Sfxn3 | Glrx5 |
| Mesd | Smurf1 | Oxsr1 | Rap1a | Slc6a15 | Ppp1cb | Hnrnpa2b1 | Egfr | Myo1e | Glrx3 |
| Hsd17b12 | Ublcp1 | Hpx | Snx1 | Flnc | Ldah | Rap2a | Cd47 | Cmbl | Ube2v2 |
| Lancl1 | Tm9sf4 | Eif3l | Rpl23 | Trim28 | Apoc1 | Asah1 | Ddx17 | Pvalb | Dynlrb1 |
| Rps13 | Efemp1 | Rps10 | Yars1 | Gmps | Ttr | Vapa | Mt3 | Atp5mc1 | Acsl3 |
| Marcks | Septin11 | Apoa2 | Tbc1d13 | Gemin5 | Aldh6a1 | Pcyox1l | Nif3l1 | Arl8b | Ak3 |
| Erlin2 | Ndrg1 | Hs2st1 | Ptma | Aldh1a1 | Synj1 | Ndufs2 | Itga6 | Ykt6 | Hook3 |
| Clec2l | Abcb7 | S100a9 | Rab18 | Impdh2 | Pccb | Rab11fip5 | Tmsb10 | Uap1l1 | Ndufs5 |
| Actbl2 | Metap2 | Fdps | C3 | Pfdn6 | Acbd3 | Phf5a | Pspc1 | Carhsp1 | Ak2 |
| Jpt1 | Tom1 | Cab39 | Ptgfrn | Hnrnpa3 | Txndc5 | Vkorc1l1 | Entpd1 | Emc6 | Ghitm |
| Spr | Slc25a22 | Gba1 | Rpl37a | Arrb1 | Ckm | Lrsam1 | Usp19 | Atp5pd | Fbxl20 |
| Stxbp1 | Gdap1 | Fyn | Acat1 | Mrc1 | Acsl1 | Gsk3b | Mapk11 | Niban2 | Cbx5 |
| Tmx4 | Aars1 | Sec63 | Timm9 | Atp2a1 | Fuom | Setd3 | Nefh | Ephx1 | Park7 |
| Lifr | Golm2 | Lgals3 | Actn1 | Dpysl4 | Plcb3 | Drg1 | Septin8 | Ywhab | Tars3 |
| Glrx | Pdcd6 | Ppp2ca | Epb41l3 | Gstz1 | Gpx4 | Mrpl1 | Eif2b4 | Mfsd10 | Timm23 |
| Cdc37 | Pcbp3 | Lnpk | Sptbn1 | Pzp | Rida | Ctsz | Eno3 | Hid1 | Pfdn5 |
| Mat2a | Acat2 | Hexa | Rab6a | Maneal | Kars1 | Acsl6 | Ubqln1 | Pam16 | Apod |
| Pef1 | Pcbp4 | As3mt | Rab5c | Armc6 | Gc | Dmtn | Uqcrfs1 | Dnajc19 | Kctd12 |
| Gjc3 | Reep1 | Ctsl | Rab21 | Mipep | Lmnb2 | Khsrp | Tmem33 | Coro7 | Akap12 |
| Tnpo1 | Hsd17b7 | Pacsin2 | Cops5 | Aldh5a1 | Sh3bgrl3 | Cops4 | Slc25a11 | Map1lc3b | Cs |
| Cybc1 | Col18a1 | Mybbp1a | Camk1d | Cars1 | Man2c1 | Cops6 | Pon2 | Tmem186 | Rps20 |
| Ivd | Samm50 | Tspan8 | Rps26 | Gan | Nmt1 | Cops3 | Ndufb7 | Lsamp | Scg3 |
| Pirt | Nqo2 | Ambp | Emc1 | Aspa | Hsd17b11 | Dab2 | Txndc12 | Rab14 | Ddx19a |
| Gls | Grb2 | Eepd1 | Rps28 | Ube2o | Gdpd1 | Fubp1 | Golt1b | Hgs | Ddx5 |
| Rhoa | Mbnl2 | Fkbp15 | Hibch | Rpl10a | Epb41l2 | Uba6 | Esyt1 | Tlcd3b | Lxn |
| Cisd3 | Flot2 | Crk | Rps25 | Set | Farsb | Itgb1 | Rer1 | Hmgn2 | Plxna1 |
| Scamp1 | Mtch1 | Etnk1 | Erap1 | Hspg2 | Itgb5 | Safb | Ptdss1 | Etfb | Coq6 |
| Purg | Rtn3 | Raly | Vps35 | Shisa4 | Fam3c | Ppt1 | Srsf2 | Txndc17 | Poldip2 |
| Acsl4 | Atp5f1a | Cisd1 | Plcd1 | Lgals1 | Atxn2 | Rbmx | Ddx3y | Ogn | Trpv1 |
| Naa25 | Rtn4 | Selenom | Psmd1 | Fads1 | Pfkp | Vwa1 | Psma5 | Cyb5r3 | Adh1 |
| Oplah | Isyna1 | Atp9a | Pygb | Micos13 | Ubl7 | Ppme1 | Gmppb | Pfkl | Atp5f1d |
| Fnbp1l | Dnm1 | Icmt | Apoa4 | Spock2 | Grk2 | Shoc2 | Psma7 | Ap1b1 | Ociad1 |
| Lpp | Ldha | U2af2 | Syp | Setd7 | Akt3 | Gabbr1 | Rmdn1 | Ilvbl | Pfkm |
| Tpd52 | Dync1h1 | Ppp2r2a | Tmem256 | Ckb | Pip4p1 | Mcam | Grn | Iars1 | Emc2 |
| Atad1 | Palmd | Rras | Bcat2 | Myh10 | Skp1 | Ptrh2 | Ddx46 | Eef1a2 | Lonp1 |
| Ppia | Rbfox2 | Ddb1 | Gsn | Fus | Cul1 | Slc7a1 | Acp1 | Agpat4 | Epdr1 |
| Agps | Extl2 | Mycbp | Enpp5 | Ostc | Tm9sf2 | Rps3 | S100a13 | Ubl3 | Dapk1 |
| Eef2 | Atp6v1d | Copa | Plp1 | Amph | Pcbp1 | Lrpprc | Rpl14 | Srm | Vkorc1 |
| Etf1 | Ctbp1 | Rab2b | Cert1 | Abcb1a | Pcnp | Preb | Mri1 | Pmm2 | Memo1 |
| Rdh14 | Myl6 | Acads | Rpl3 | Cadm4 | Lmna | Cfl2 | Atp6v1g1 | Grsf1 | Mras |
| Thop1 | Npm1 | Timm8a1 | Elob | Igkc | Parva | Gpx1 | Dnajc11 | Ethe1 | Lypla2 |
| Snx4 | Mlec | Bub3 | Wdr1 | Eif5a | Cavin3 | Abhd6 | Mrpl4 | Bdh1 | Mapk1 |
| Kng1 | Erp29 | Tagln2 | Slc4a4 | Plaat3 | Srp68 | Prkag2 | Pllp | Gstk1 | Ppp1cc |
| Zzef1 | Cyrib | Yipf5 | Timm44 | Thy1 | Idi1 | Cntfr | Sec61b | Ppib | Sdcbp |
| Hrg | Mccc2 | Fermt2 | Marchf5 | Fbln5 | Lamb1 | Rpl31 | Nucks1 | Anxa3 | Atp1a2 |
| Atp5f1b | Dbt | Slc16a1 | Tpm2 | Cst3 | Hnrnpul2 | Pgp | Slc25a46 | Vamp7 | Cdk5 |
| Smpd1 | Eif4b | Ldhb | Tmem109 | Pxn | Rpl9 | M6pr | Cast | Ppp1r2 | Cirbp |
| Slc25a40 | Nek7 | Agap3 | Slc4a1 | Crip1 | Me1 | Stbd1 | Pdcd5 | Paics | Ruvbl2 |
| Asl | Prkcb | Ncald | Cav1 | Stxbp6 | Svip | Atp6v1h | Crip2 | Aldh1l1 | Atox1 |
| Sorbs1 | Hcn1 | Rab2a | Pgm2l1 | Ncs1 | Enpp1 | Impdh1 | Ywhaz | Sec62 | Serpina3m |
| Cfd | Irgq | Ppic | Vim | Psmb8 | Rnh1 | Rps7 | Gars1 | Sub1 | Itgb3 |
| Dbnl | Nudt3 | Anapc1 | Pitpnm2 | Ptp4a2 | Bcas1 | Bcr | Hsdl1 | Psmb5 | Ube2v1 |
| Hspb1 | Lamtor2 | Slc25a51 | Dhcr7 | Psmd13 | Ranbp1 | Timm8b | Micos10 | Slc30a9 | Hsph1 |
| Capn5 | Clpx | Fkbp4 | Ighm | Txn2 | Sri | Snx6 | Dnpep | Ndufb9 | Tubb5 |
| Pigt | Cpne1 | Ehd4 | Tmod1 | Gclc | Ogt | Upf1 | Acot7 | Pdlim4 | Rplp2 |
| Pck2 | Prepl | Cnbp | Ogdh | Smarcc1 | Dag1 | Mccc1 | Ndufs3 | Bcl2l13 | Vcl |
| Synj2bp | Rpl24 | Entpd5 | Cttn | Ube2i | Ddx3x | Timm13 | Rtcb | Tpd52l2 | Psmb4 |
| Gnas | Golga2 | Eif2b5 | Prnp | Rps17 | Rab3gap2 | Rps27l | Coa3 | Prkar2a | Prdx5 |
| Snd1 | Sf3b3 | Ano6 | Orm1 | Prdx3 | Slc25a32 | F2 | Acot13 | Sigmar1 | Atp1a3 |
| Uqcrh | Gng3 | H2ac20 | Vmp1 | Gga1 | Rps19 | Atp5mg | Gsta4 | Cisd2 | Fis1 |
| Hspa1a | Tmem43 | Pgam1 | Nsfl1c | Atg3 | Pip4p2 | Fhl1 | Abca9 | Sccpdh | Rpe |
| Emc4 | Wars1 | Ndufaf4 | Ndrg4 | Sec31a | Gart | Anpep | P2rx7 | Nedd4l | Ndufa3 |
| Serpina1b | Erp44 | Mtpn | Naxd | Uqcr11 | Slc27a4 | Tomm22 | Usp9x | Glg1 | Nol3 |
| Cr1l | Mpz | Tbca | Pgd | Dpp3 | Ndufs1 | Lamtor1 | Hnrnpl | Ewsr1 | Cmas |
| Tmed4 | Cox6c | Ndufs6 | Pycr3 | Anxa11 | Gpm6b | Vwa5a | Rpl18a | Slc25a3 | Uqcrc1 |
| Gnaq | Txnrd2 | Vps29 | Ap3b2 | Rpl12 | S100a10 | Mcrip1 | Hagh | Sorl1 | Vps45 |
| Eif4a1 | Fga | Vcp | Snap91 | Gpd1 | Gpm6a | Cpne3 | Lamtor4 | Ntpcr | Rplp1 |
| Gna11 | Stt3b | Ruvbl1 | Ssbp1 | Stard10 | Clu | Ddx39b | Plg | Rpl5 | Actr10 |
| Fth1 | Sfxn5 | Lrrc57 | Snx27 | St13 | Pcp4 | Syvn1 | Mag | Rps4x | Paqr9 |
| Alad | Ndufa5 | Hhatl | Sel1l | Pip4k2a | Myo1c | Timm22 | Hint1 | Rpl13 | Ppif |
| Sult4a1 | Atp5pf | Pmvk | Rab11b | Eif2s2 | Rpl15 | Arpc5 | Ipo5 | Aamdc | Ckmt1 |
| Hspa2 | Chordc1 | Abca8b | Tial1 | Cds2 | Stom | Crmp1 | Higd1a | Eea1 | Lactb2 |
| Grpel1 | Chmp3 | Mtdh | Hspa5 | Rpl18 | G6pc3 | Cuta | Cmpk1 | Gdi2 | Cops7a |
| Vamp2 | Abcb6 | Cbx3 | Sdhb | Pkm | Eif4e | Anxa4 | Sec61a2 | Arhgdib | Crkl |
| Eprs1 | Slc36a1 | Ergic1 | Fasn | Ptdss2 | Arhgap17 | Glod4 | Nfu1 | Capg | Cplx2 |
| Acly | Prxl2a | Uba2 | Flna | Ldlr | Tppp3 | Mmab | Col14a1 | Acaca | Erh |
| Serpinf1 | Cntnap2 | Mrpl21 | Ndufa8 | Tcp1 | Rac1 | Ndufa2 | Nebl | Eml2 | Sod2 |
| Dpep1 | Susd2 | Ctnna1 | Ssb | Dpm1 | Chchd3 | Emc3 | Cd2ap | Stard3nl | Prpf19 |
| Gpaa1 | Auh | Atp6v0a1 | Syngr3 | Hnrnpu | Plpbp | Tomm34 | Sec13 | Ero1a | Ank1 |
| Ndufb3 | Dctn2 | Hnrnph2 | Slc25a35 | Ap2m1 | Mdh2 | Aco2 | Ufd1 | Eif3a | Spcs2 |
| Ndufa6 | Hnrnpc | Tubb2a | Akr1b1 | Tmem30a | Tsfm | Limch1 | Eef1e1 | Psma2 | Crat |
| Itgav | Flnb | Atp6v1c1 | Ndufb5 | Arf5 | Dync1li1 | Ndufa7 | Rack1 | Dlst | Atp1b3 |
| Cdk5rap3 | Fahd1 | Rgs3 | Akr1b8 | Atp6ap2 | Dpy30 | Nat8l | Nt5c | Hdhd3 | Cd200 |
| Hmgcl | Pabpc1 | Ppfia2 | Btf3l4 | Oxr1 | Pacsin1 | Dpp6 | Chil3 | Hprt1 | Psme1 |
| Flot1 | Uqcrq | Alg10b | Fdxr | Psme2 | Atp2b1 | Abhd16a | Babam2 | Fxr1 | Eno1 |
| Faah | C2cd2l | Ube2l3 | Pdhx | Slc25a10 | Apoe | Clic1 | Rhob | Enpp4 | Hspd1 |
| Aip | Ndufb2 | Map1a | Hexb | Nt5e | Tpt1 | Abcg2 | Hpcal1 | Cntnap1 | Mtx1 |
| Golph3 | Decr1 | Acot2 | Eif3f | Usp14 | Bcs1l | Mydgf | Hnrnph1 | Cmtm6 | Hint3 |
| Msmo1 | Glo1 | Lamtor5 | S100a4 | Eif3i | Sod1 | Ndufc2 | Prkcd | Nipsnap3b | Dhx9 |
| Emc7 | Pgls | Sgcd | Pcyox1 | G3bp2 | Prkaa1 | Ndufa9 | Ap2s1 | Tmsb4x | Igf2r |
| Dmac2l | Mtap | Sgcb | Crym | Acadl | Cplx1 | Pmpca | Nfasc | Rgs10 | Tmed9 |
| Rab3a | Mta1 | Srsf4 | Get3 | Hibadh | Abhd12 | Bag6 | Nacad | Copg1 | Arpc3 |
| Slc25a20 | Mgst3 | Chchd2 | Aak1 | Uso1 | Dnaja1 | Hsp90aa1 | Srsf7 | Znrd2 | Cul3 |
| Hspa8 | Cdc42ep4 | Syncrip | Bphl | Strap | Mpc1 | Atp6v1f | Srrm2 | Mapt | Gnai3 |
| Sacm1l | Ndufs7 | Atp1b2 | Nudt21 | Hmgb1 | Map2k4 | Gapdh | Fcgrt | Dhrs1 | Crot |
| Psph | Stk24 | Endod1 | Eif4g3 | Arf1 | Tlcd4 | Coasy | Txnrd1 | Scrn3 | Hdhd2 |
| Eci1 | Vars1 | Qki | Pip4k2b | Abracl | Eno2 | Cpne2 | Mtmr9 | Stat1 | Bag3 |
| Nfs1 | Itfg1 | Sumo1 | Nme1 | Edf1 | Stmn1 | Pitpna | Ndufs4 | Apc2 | Rpsa |
| Il6st | Ctps2 | Rpl29 | Calr | Sbspon | Rhog | Nt5dc3 | Hdgfl3 | Mblac2 | Dnajb11 |
| Acadsb | Sntb1 | Dynll1 | Vsnl1 | Arpc4 | Rpl19 | Fmr1 | Gstm1 | Dnajc5 | Stat3 |
| Rpl23a | Stim1 | Erlec1 | Mrpl14 | Bscl2 | Ces1c | Guk1 | Taldo1 | Gpd2 | Pigk |
| Rps6 | Fscn1 | Arl8a | Mcee | Ndufb4 | Eif3d | Mcub | Nid2 | Sar1b | mt-Co3 |
| Elavl1 | Rab5a | Banf1 | Pdcd10 | Scp2 | Lap3 | Ecsit | Septin2 | Eif3k | Huwe1 |
| Sarnp | Apeh | Gpc1 | Ilf2 | Ctnnal1 | Abhd11 | Dclk1 | Adss2 | Rpl6 | Card19 |
| Luc7l2 | Cryab |  |  |  |  |  |  |  |  |

*Supplement Figure 1*: Venn diagram showing common DRG proteins between our dataset and other mouse models of neuropathy. 88% of our DRG proteome (2795 proteins) is found to be shared with another DRG proteome within a mouse model for pain research by Pogatzki-Zahn et al., 2021.


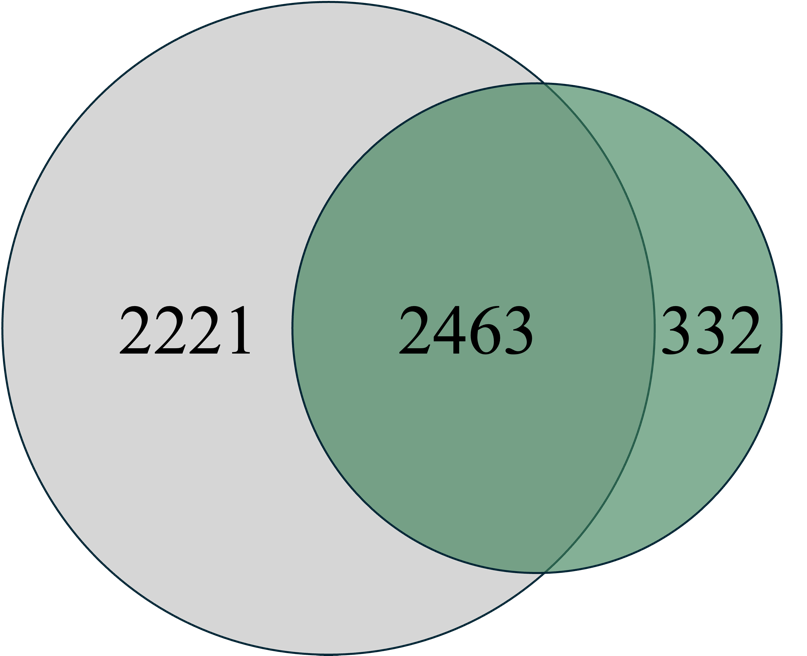


**88%**
